# Molecular profiling and functional analyses of umbilical cord mesenchymal stem cell-derived extracellular vesicles for dry eye diseases

**DOI:** 10.20517/evcna.2025.177

**Published:** 2026-06-16

**Authors:** Qiao-Yu Hsu, Chia-Ni Hsiung, Hsin-Hung Cheng, Wen-Yu Lien, Wen-Hsien Lin, Martin Sieber, Chi-Chih Kang

**Affiliations:** ^1^BIONET Therapeutics Corp., Taipei 114065, Taiwan.; ^2^Genetics Generation Advancement Corp. (GGA Corp.), Taipei 114065, Taiwan.; ^3^BIONET Corp., Taipei 114065, Taiwan.; ^#^These authors contributed equally to this work.

**Keywords:** Umbilical cord derived mesenchymal stem cells, extracellular vesicles, dry eye diseases, proteomic analysis, miRNA profiling, immunomodulation, regeneration

## Abstract

**Aim:** Dry eye disease (DED) is a multifactorial disorder characterized by tear film instability, ocular surface damage, and inflammation. This study investigates the molecular landscape and the therapeutic potential of umbilical cord mesenchymal stem cell-derived extracellular vesicles (UCMSC-EVs) as a standardized, cell-free treatment for DED.

**Methods:** UCMSC-EVs were produced from UCMSCs in a scalable 3D bioreactor system and enriched via tangential flow filtration (TFF). The physicochemical properties of UCMSC-EVs were characterized. The molecular cargo of UCMSC-EVs was analyzed via proteomics analysis and microRNA (miRNA) microarray profiling across multiple production lots. Functional potency of UCMSC-EVs was assessed using a human corneal epithelial cell line (HCE-S) wound healing assay and two human inflammation models.

**Results:** UCMSC-EVs exhibited a consistent cargo profile enriched with proteins and miRNAs targeting DED-related pathways, with high proteomic correlation (Pearson’s *r* > 0.8) across independent lots. Functional assays demonstrated that UCMSC-EVs promoted HCE-S wound healing and suppressed pro-inflammatory cytokines in a dose-dependent manner. Furthermore, a significant negative correlation was identified between PEDF (Pigment Epithelium-Derived Factor) cargo concentration and interferon gamma (IFN-γ) secretion (Pearson’s *r* = -0.724, *P* = 0.005), providing a quantitative link between molecular identity and anti-inflammatory potency.

**Conclusion:** UCMSC-EVs possess favorable physicochemical and molecular characteristics, and demonstrate robust regenerative and anti-inflammatory properties *in vitro*. The high manufacturing consistency and the identification of key molecular drivers, such as PEDF, support the potential of UCMSC-EV as a well-characterized and viable cell-free therapy for mitigating DED progression.

## INTRODUCTION

Dry eye disease (DED) is a chronic, progressive ocular surface disorder that affects millions globally, and significantly impacts quality of life. Characterized by a loss of tear film homeostasis, DED leads to visual disturbance, ocular irritation, neuropathic pain, and in severe cases, corneal ulceration, and conjunctival scarring^[1,2]^. Despite the prevalence of artificial tears and prescription medications for DED, patient adherence remains poor. Recent data from the American Academy of Ophthalmology’s IRIS (Intelligent Research in Sight) Registry indicate that 90% of patients discontinue their treatments (e.g., lifitegrast, cyclosporine A) within 1 year, due to limited efficacy and adverse effects^[3]^. This high attrition rate underscores an urgent need for novel therapeutics that address the complex, multimodal pathophysiology of DED.

Mesenchymal stem cell (MSC) therapies have shown immense promise in various degenerative and inflammatory ocular conditions^[4]^. In the past five years, 28 clinical trials have utilized MSCs for ocular diseases, including seven targeting age-related macular degeneration and six targeting DED^[4]^. Our recent study has demonstrated that umbilical cord-derived mesenchymal stem cells (UCMSCs) improve tear production and ocular surface integrity of patients with Stevens-Johnson syndrome and toxic epidermal necrolysis, highlighting the regenerative potential of UCMSCs in treating ocular surface diseases^[5]^. However, the clinical translation of live MSCs is hindered by safety concerns, including potential immunogenicity as well as challenges in Chemistry, Manufacturing, and Controls (CMC) standardization^[4]^.

Consequently, research has shifted toward cell-free alternatives. Established evidence shows that the therapeutic potency of MSCs is primarily mediated by extracellular vesicles (EVs), i.e., exosomes and microvesicles^[6]^. These nano-sized, lipid bilayer vesicles shuttle bioactive proteins, lipids, and microRNAs (miRNAs) to facilitate intercellular communication among cells. MSC-derived EVs (MSC-EVs) offer distinct advantages over parental cells, including reduced immunogenicity, potential for large-scale standardized production, and superior bioavailability^[7,8]^. Preclinical *in vitro* and *in vivo* models of dry eye and other ocular diseases have demonstrated that MSC-EVs promote corneal regeneration and immunomodulation by accelerating epithelial wound healing, reducing apoptosis, and suppressing inflammatory cytokine expression^[7,9]^. Our own *in vivo* study showed that topical UCMSC-EV treatment exhibited robust regenerative and immunomodulatory effects in a severe DED rat model involving lacrimal gland resection^[10]^. Specifically, UCMSC-EVs reduced the expression of pro-inflammatory cytokines, limited epithelial cell apoptosis, and suppressed fibrotic remodeling by preventing myofibroblast activation^[10]^. While the regenerative and immunomodulatory capacities of UCMSC-EVs are evident, the precise molecular mechanisms underlying these effects remained poorly understood. Identifying the specific cargo, particularly protein and miRNA players, is essential for defining critical quality attributes and establishing the specifications required for standardized manufacturing.

In this study, we integrated comprehensive proteomic and miRNA profiling with functional assays to characterize the regenerative and anti-inflammatory potency of UCMSC-EVs. The findings demonstrate that UCMSC-EVs robustly suppress inflammation and promote wound healing *in vitro*. Notably, we found that the anti-inflammatory effect significantly correlates with the protein expression of PEDF (Pigment Epithelium-Derived Factor), a key protein candidate identified through proteomic profiling and quantitatively validated via enzyme-linked immunosorbent assay (ELISA). Based on these data, we propose a mechanism of action (MoA) of UCMSC-EV involving nuclear factor kappa B (NFκB), hypoxia-inducible factor 1 alpha (HIF-1α), and interleukin-6 (IL-6) signaling alongside tissue inhibitor of metalloproteinases 1 (TIMP1)-mediated direct regulation of matrix metalloproteinase-9 (MMP-9)^[11,12]^ to mitigate DED progression. While these bioinformatics predictions warrant further experimental validation, these results establish a directional mechanistic framework for the therapeutic use of large-scale manufactured UCMSC-EV in DED.

## METHODS

### UCMSC-EV production and isolation

The UCMSCs were isolated from the Wharton’s jelly of a donated umbilical cord. The donor had signed the informed consent form, and the donor’s blood was examined to be free from human infectious diseases (i.e., HBV, HCV, HIV). The primary UCMSCs were expanded to generate the cell bank, which was examined to show stem cell markers, including CD13, CD29, CD44, CD73, CD90, and CD105, while lacking the expression of hematopoietic and endothelial markers CD14, CD19, CD31, CD34, CD45, and HLA-DR by flow cytometry^[13]^. In addition, the stemness of UCMSCs was confirmed by successful osteogenic, adipogenic, and chondrogenic differentiation under lineage-specific induction conditions^[13]^. All the lots generated in this study are from the same donor.

For UCMSC-EV production, the UCMSCs at passage 5 were inoculated into the bioreactor (Scale-X Hydro system, Univercells Technologies, Belgium). Once reaching the desired confluence, the UCMSCs were washed and changed to the serum-free and xeno-free α-MEM (Minimum Essential Medium) media (no phenol red, 41061-029, Gibco) for EV collection. After 4 days, twenty liters of collected conditioned media were clarified with a continuous centrifuge (UFMini, Carr Biosystems, USA) at 4,000 × *g* to eliminate cells and debris. Then, the clarified media were concentrated by tangential flow filtration (TFF) (DP-01 TFF system, LEF Science, Taiwan). The EV-enriched solution was further filtered through a 0.2 μm membrane filter (AseptiCap, MDI membrane, USA). The final solution of UCMSC-EVs was stored at -80 °C in small aliquots for further use.

### Nanoparticle tracking analysis

The size distribution and concentration of UCMSC-EVs were measured using the ZetaView PMX 130 system (Particle Metrix, Germany) and analyzed with ZetaView software, following manufacturer’s guidelines. Prior to measurement, the UCMSC-EVs were diluted in Dulbecco’s phosphate-buffered saline (DPBS, 14190144, Gibco) to the concentration range recommended by the manufacturer.

### Flow cytometric characterization of exosomal surface markers

For surface marker analysis, UCMSC-EVs were captured using the Exosome-Human CD81 Flow Detection Reagent (10622D, Invitrogen^TM^) and subsequently stained with PE-conjugated antibodies against CD9 (555372), CD63 (556020), and CD81 (555676) (BD Pharmingen). A PE-conjugated IgG1κ isotype control (555749, BD Pharmingen) was included for background assessment and gating. All samples were analyzed using a FACSLyric^TM^ flow cytometer (BD Pharmingen, Canada).

### Cryo-electron microscopy (Cryo-EM)

Quantifoil R1.2/1.3 300-mesh grids coated with a 2 nm continuous carbon film were glow-discharged for 80 s at 30 mA using a GloQube Plus system (Quorum, UK). A 4 µL aliquot of the UCMSC-EV sample was applied to the grid and blotted for 2 s using a Vitrobot Mark IV (Thermo Fisher, USA) at 4 °C, 100% humidity. The grids were plunged into liquid ethane, cooled by liquid nitrogen. Data were acquired using EPU software on a Glacios Cryo-EM (Thermo Fisher, USA), operating at 200 kV with a Falcon 4 detector. Images were acquired at 92,000× magnification in counting mode (1.6 Å/pixel), and Feret’s diameter was analyzed using ImageJ (NIH, USA).

### UCMSC-EV lipidomic analysis

Lipids were extracted from two UCMSC-EV lots using a modified Folch lipid extraction method. The extracted sample was reconstituted with 200 μL of methanol. Finally, a 5 μL aliquot of the sample was injected into the LC-MS/MS system. This analysis was performed using an Agilent 1290 UHPLC system (Santa Clara, USA) coupled to an Agilent 6545XT AdvanceBio LC/Q-TOF. A ZORBAX Eclipse RRHD Plus C18 analytical column (1.8 μm; 2.1 mm × 100 mm, Agilent) was used for the separations, and the column temperature was maintained at 55 °C. The mobile phase consisted of 10 mM ammonium acetate in 40% acetonitrile (phase A) and 10 mM ammonium acetate in 10% acetonitrile and 90% isopropanol (phase B), with a flow rate of 0.35 mL/min. The Q-TOF was operated in Auto MS/MS mode, with a mass range of 100-1,700 for MS and MS/MS. The LC-MS/MS analysis was performed with both positive and negative ion modes. Four technical replicates were analyzed for each mode. Lipids were identified using MSDIAL ver.5.1.230807 (RIKEN Center for Sustainable Resource Science). The identification was based on the precursor m/z accuracy and MS/MS spectra. Peak areas and alignments were performed using MSDIAL. Data were manually inspected. Incorrectly identified information and signals with a relative standard deviation exceeding 20% were excluded. Lipid quantification was performed by comparing the peak area of the target lipid with the corresponding SPLASH® LIPIDOMIX® Mass Spec Standard.

### UCMSC-EV proteomic analysis

The protein cargo of the UCMSC-EVs was analyzed using the LC-MS/MS mass spectrometry-based proteomics. The UCMSC-EVs were lysed to extract proteins. The proteins were digested overnight with trypsin at a ratio of 1:50 (w:w trypsin:protein). The digested peptide was desalted using a C18 column before the LC-MS/MS analysis. The peptides were separated in the mobile phase A (0.1% formic acid in water) and the mobile phase B (100% acetonitrile with 0.1% formic acid) within 90 min at a flow rate of 300 nL/min followed by analysis on an Orbitrap Fusion Lumos Tribrid quadrupole-ion trap-Orbitrap mass spectrometer (Thermo Fisher Scientific, San Jose, Canada) with a resolution of 15,000 within a 1.4 Da isolation window. The LC-MS/MS data was quantified using Proteome Discoverer 2.5 with FDR < 1%. MaxQuant software package (v1.6.6.0) was used to retrieve the secondary MS data, and the Swiss-Prot_Human data was used as reference (20,600 proteins; Proteome ID: UP000005640).

To identify consistently abundant proteins across lots, proteins with non-zero normalized abundance were ranked independently within each UCMSC-EV lot, and the top 10% most abundant proteins were selected per lot. Only proteins shared among the top 10% in all three lots were retained for downstream analyses. Pathway and regulatory network enrichment analyses of this intersecting protein set were performed using Ingenuity Pathway Analysis (IPA) (IPA; QIAGEN Inc.)^[14]^, a knowledge-based analysis of curated literature to map molecules to pathways and functions. IPA analysis identifies enriched canonical pathways as well as upstream and downstream regulators of selected key protein candidates. Statistical significance for the identified pathways was determined using a Fisher’s exact test, with results filtered by a *P*-value < 0.05 to prioritize biologically relevant results. This bioinformatic approach constructed a network, linking UCMSC-EV protein cargoes to the immunomodulation and wound healing functions. These pathways were visualized using dot plots, generated by ggplot2 R package. In addition, the Pearson’s correlation among the three UCMSC-EV lots was calculated using the corrplot R package to assess lot-level similarity. The statistical significance of the Pearson’s correlation was determined by one-sample *t*-test based on the Pearson correlation coefficient (*r*).

### UCMSC-EV protein quantified by ELISA analysis

The proteomically identified candidates, PEDF and FN1 (Fibronectin), were quantified by PEDF ELISA (ab246535, Abcam) and Fibronectin ELISA (ELH-FN1-1, RayBiotech), respectively. The ELISA was executed following the manufacturer’s instructions. Absorbance at 450 nm was recorded using the SpectraMax iD3s Multi-Mode Microplate Readers (Molecular Devices, USA). After subtracting the blank values, the sample concentrations were interpolated based on the standard curve.

### UCMSC-EV miRNA microarray analysis

Amniotic fluid (AF), cord blood plasma (CBP), platelet and UCMSC derived EV samples were collected from BIONET Corp. All the donors had signed the informed consent forms. Total miRNA was extracted using the miRNeasy Serum/Plasma Kit (217184, QIAGEN), and RNA concentration was measured by “SpectraMax iD3 Multi-Mode Microplate Reader” (Molecular Devices, USA). Samples were stored at -80 °C until use. For microarray analysis, miRNAs were labeled with the FlashTag^TM^ Biotin HSR RNA Labeling Kit (902922, Thermo Fisher Scientific) according to the manufacturer’s instructions, including poly(A) tailing and biotin ligation steps. RNA Spike Control Oligos were used to monitor labeling and hybridization efficiency. Labeled RNA was hybridized to the GeneChip^TM^ miRNA 4.0 Array (902411, Thermo Fisher Scientific) at 48 °C for 16-18 h, followed by washing and staining on the GeneChip® Fluidics Station 450 and scanning with the GeneChip® Scanner 3000.

The miRNA microarray data (Raw .CEL files) were processed using the R packages oligo and pd.mirna.4.0 to read the CEL files into expression count data. Quality control (QC) and normalization were subsequently performed within the R environment to ensure data reliability and comparability across samples. Predicted target mRNAs for the identified miRNAs were then analyzed using IPA (QIAGEN, Canada). In IPA, a Fisher’s exact test was applied to evaluate the probability of the predicted mRNAs being associated with specific canonical pathways compared to random distribution. The significance of pathway enrichment was represented by the *P*-value, where lower *P*-values (displayed as higher values in the table after -log(P) transformation) indicate stronger associations between the miRNA-predicted mRNAs and the corresponding pathways. These pathways were visualized using dot plot, generated by ggplot2 R package.

### Corneal epithelial wound healing assay

Immortalized human corneal epithelial (HCE-S) cells were obtained from ABM Inc. (T0737, ABM Inc). Cells were maintained in PriGrow III medium (TM003, ABM Inc.) supplemented with 10% fetal bovine serum (FBS, A5256701, Gibco), 1X L-Glutamine (SH30034.01, HyClone, USA), and 1% Antibiotic-Antimycotic (100X) (15240062, Gibco). For the wound healing experiments, HCE-S cells were seeded into 6-well plates at 1 × 10^6^ cells per well and grown to confluence after 2 days. The culture medium was replaced with 10 μg/mL mitomycin C (ROC-10107409001, Roche) in PriGrow III medium and incubated at 37 °C for 3 h. After mitomycin C treatment, the cells were washed with DPBS, and a scratch wound was created using a sterile P1000 pipette tip. The cells were imaged using the Mateo TL microscopy (Leica, Germany) to acquire the baseline images. Cells were then treated with varying particle concentrations of UCMSC-EVs in the α-MEM media. After 24 h, the wound closure was imaged again and quantified using ImageJ. The cell-free area or wound area at 24 h for various UCMSC-EV concentrations was normalized to that at t_0_ using the following equation^[15]^.

**Figure:**
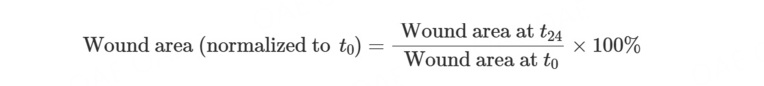


The wound area, normalized to t_0_, data was analyzed with the four-parameter logistic curve fitting (GraphPad Prism 10, GraphPad Software, USA) to show dose-response relationship.

### Lipopolysaccharide-induced THP-1 monocyte inflammatory assay

The THP-1 cells were obtained from Bioresource Collection and Research Center (BCRC, 60430, Taiwan). Cells were maintained and grown in RPMI 1640 medium (A1049101, Gibco) supplemented with 10% FBS (A5256701, Gibco), and 1% Antibiotic-Antimycotic (100X) (15240062, Gibco). THP-1 cells were seeded into 6-well plates at 2 × 10^6^ cells per well. Upon attachment, THP-1 cells were cultured in medium containing 100 ng/mL phorbol-12-myristate-13-acetate (PMA, 10008014, Cayman) at 37 °C in a CO_2_ incubator for 24 h to induce macrophage-like differentiation. The differentiated THP-1 cells were washed with DPBS, and cultured in the complete media for 24 h. Following recovery, the THP-1 cells were washed with DPBS and then induced with 0.1 µg/mL lipopolysaccharide (LPS, SI-L6529, Sigma) in the presence or absence of different concentrations of UCMSC-EVs for 24 h. The gene expression (GE) of the induced inflammatory cytokines (IL-6, IL-1β, TNF-α) were quantified by reverse transcription quantitative polymerase chain reaction (RT-qPCR). Total cell RNA was extracted using Quick-RNA^TM^ MiniPrep Kit (R1055, ZYMO Research) following the manufacturer’s instructions. The RT-qPCR was performed with Power SYBR^TM^ Green RNA-to-CT^TM^ 1-Step Kit (4389986, Thermo Fisher) on QuantStudio^TM^ 5 Real-Time PCR System (Thermo Fisher, USA). The relative expression of all mRNAs was normalized to GAPDH. The data were calculated using the 2^-ΔΔCt^ method. The primer sequences are detailed in Table 1.

**Table 1:** Forward and reverse primers used in the inflammatory cytokine RT-qPCR study

**Primer**	**Primer sequence (F)**	**Primer sequence (R)**
Hu_GAPDH	GCACCAGGTGGTCTCCTCT	TGACAAAGTGGTCGTTGAGG
Hu_IL-1β	CCACAGACCTTCCAGGAGAATG	GTGCAGTTCAGTGATCGTACAGG
Hu_IL-6	GGTACATCCTCGACGGCATCT	GTGCCTCTTTGCTGCTTTCAC
Hu_TNF-α	CTCTTCTGCCTGCTGCACTTTG	ATGGGCTACAGGCTTGTCACTC

RT-qPCR: Reverse transcription quantitative polymerase chain reaction; GAPDH: glyceraldehyde-3-phosphate dehydrogenase; IL-1β: interleukin-1beta; IL-6: interleukin-6; TNF-α: tumor necrosis factor-alpha.

The relative cytokine GE at different UCMSC-EV concentrations was calculated by normalizing the GE of the negative no-stimulation control and LPS-induced samples using the following equation^[16]^.

**Figure:**
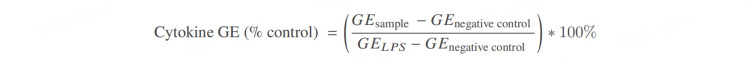


Lower relative cytokine GE indicates higher anti-inflammatory potency of the UCMSC-EVs. The data were analyzed with the four-parameter logistic curve fitting (GraphPad Prism 10, GraphPad Software, USA) to show dose-response relationship. The top of the curve (maximum response) was defined by the LPS control, whereas the bottom (minimum response) was defined by the negative (no-stimulation) control.

### PHA-induced peripheral blood mononuclear cells inflammatory assay

Peripheral blood mononuclear cells (PBMCs) were isolated from human peripheral blood using Leucosep tube (227288, Greiner) according to manufacturer’s instructions. The isolated PBMCs were cultured in α-MEM medium (12571071, Gibco) supplemented with 30% human AB serum (Access Biologicals, USA). The PBMCs were seeded into 24-well plates at 2 × 10^5^ cells per well and induced with 10 µg/mL PHA (SI-L8754-5, Sigma) in the presence or absence of UCMSC-EVs for 72 h. After incubation, the supernatants were collected for interferon gamma (IFN-γ) ELISA analysis. The IFN-γ ELISA (ELH-IFNg, RayBiotech) was executed following the manufacturer’s instructions.

### Statistics

Quantitative data are presented as the mean ± standard deviation (SD), with error bars in all figures representing the SD of independent replicates. For functional efficacy assays (*n* = 3 independent replicates), statistical significance was determined using one-way ANOVA followed by Dunnett’s post-hoc test for multiple comparisons against the control group. Concentration-response curves (*n* = 2 independent replicates) were generated to characterize dose-dependent potency trends. Statistical significance was defined as *P* < 0.05. All statistical analyses were performed using GraphPad Prism 10 (GraphPad Software, USA), and specific sample sizes and *P*-values are detailed in the corresponding figure legends. The relationship between specific EV cargo (PEDF) and anti-inflammatory bioactivity was evaluated using Pearson’s correlation analysis, with the *r* and associated *P*-value reported.

## RESULTS

### Physicochemical characterization of UCMSC-EVs

**Figure 1:**
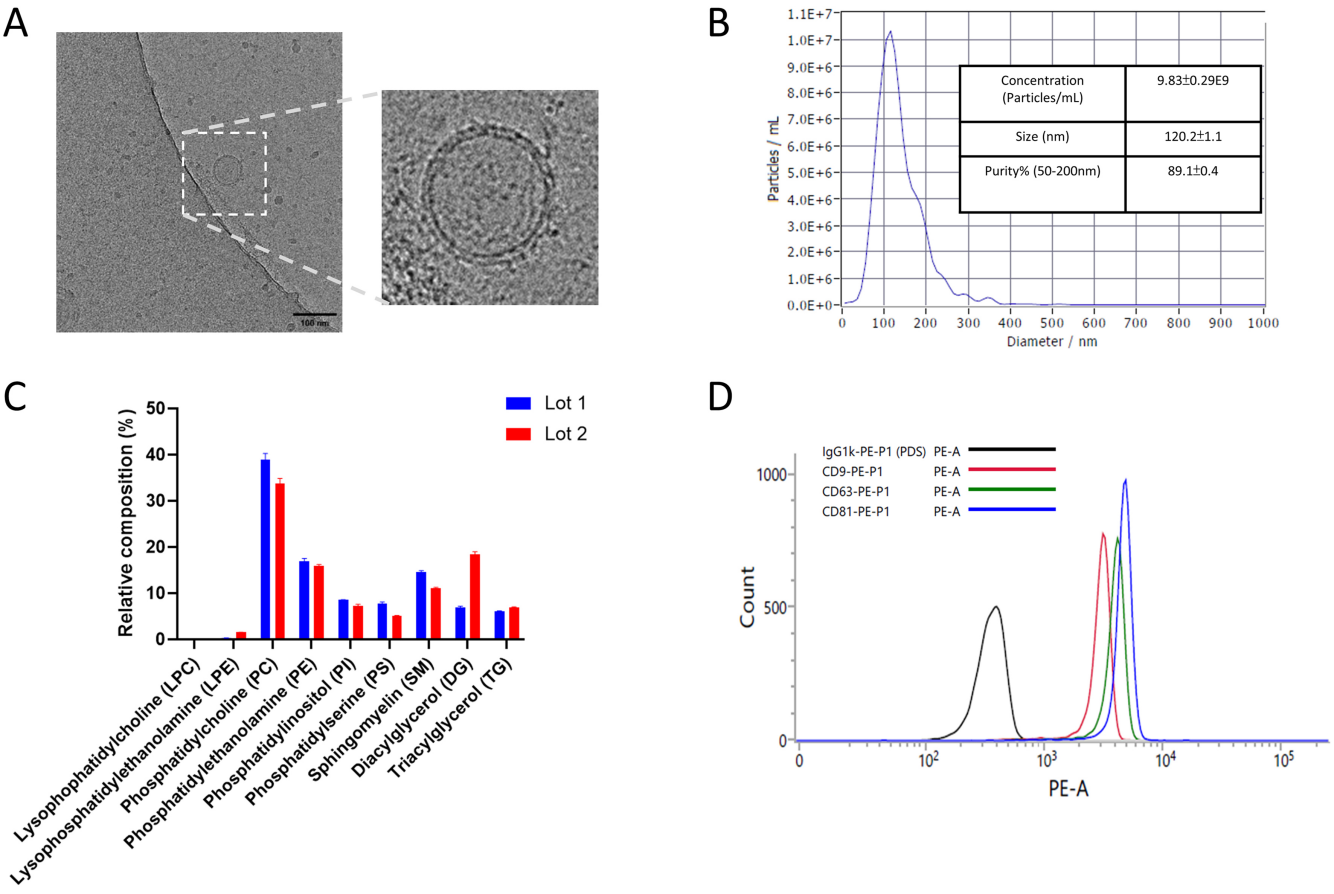
The physicochemical properties of the UCMSC-EVs. (A) A representative Cryo-EM image showed that UCMSC-EVs contained lipid bilayered particles. The scale bar is 100 nm; (B) NTA analysis showed that UCMSC-EVs had a median particle size around 120 nm with a purity of approximately 89%; (C) The bar plot shows similar lipid profiles of two independent UCMSC-EV lots measured by LC-MS/MS. Relative composition was calculated as (specific measured lipid/ total measured lipid) × 100%. The error bar is the standard deviation of four independent technical replicates of the same lot; (D) The anti-CD81 bead-based flow analysis shows that UCMSC-EVs stained positive for exosome markers (i.e., CD81, CD9, CD63), compared with the IgG isotype control. CD9: Cluster of differentiation 9; CD63: cluster of differentiation 63; CD81: cluster of differentiation 81; Cryo-EM: cryo-electron microscopy; IgG: immunoglobulin G; LC-MS/MS: liquid chromatography-tandem mass spectrometry; NTA: nanoparticle tracking analysis; UCMSC-EVs: umbilical cord mesenchymal stem cell-derived extracellular vesicles.

The UCMSC-EVs exhibited characteristic extracellular vesicle properties, appearing as intact, bilayered spherical structures under Cryo-EM [Figure 1A]. Nanoparticle Tracking Analysis (NTA) confirmed a consistent particle size distribution with a mean diameter of approximately 120 nm [Figure 1B]. Lipidomic profiling revealed a composition typical of biological membranes, with phosphatidylcholine (PC) as the primary component (~40%) [Figure 1C]. Furthermore, the presence of canonical exosome markers (CD9, CD63, and CD81), validated via bead-based flow cytometry, confirmed the successful enrichment of extracellular vesicles throughout the manufacturing process [Figure 1D]

To elucidate the molecular basis for the therapeutic activity observed in our *in vivo* models^[10]^, we performed comprehensive proteomic and miRNA microarray analyses. This multi-omics approach aimed to identify the distinct cargo profiles of UCMSC-EVs - specifically the protein and miRNA candidates likely to mediate the regenerative and immunomodulatory signaling pathways critical to DED disease progression.

### Proteomic profiling of UCMSC-EVs identifies consistently expressed proteins linked to DED signaling

**Figure 2:**
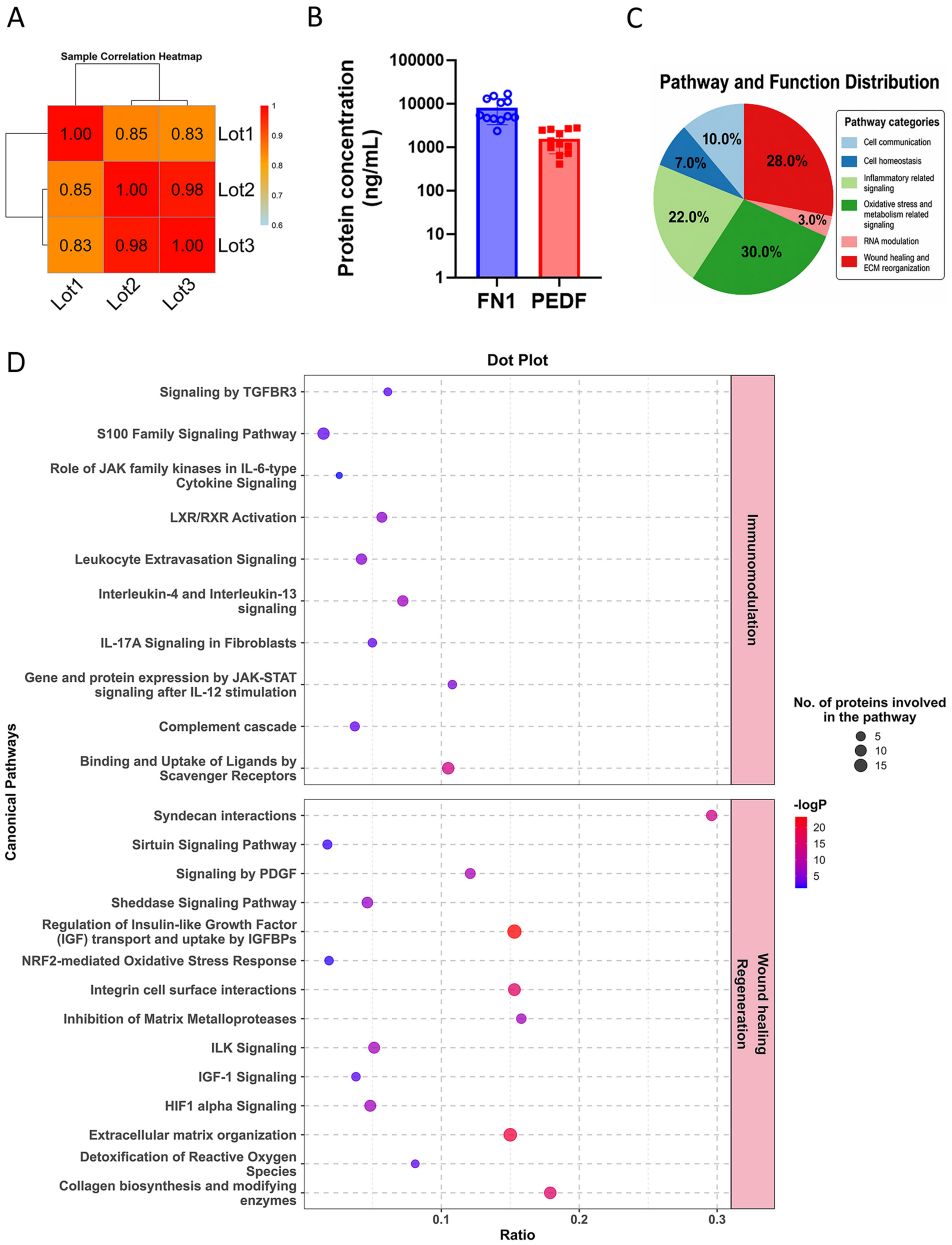
UCMSC-EV proteomic analysis showed lot-to-lot similarity and distinct proteins related to DED signaling. (A) Pearson’s correlation analysis suggested similar protein profiles among 3 UCMSC-EV lots from a single donor. Strong Pearson’s correlation coefficient (*r* > 0.8) indicates highly correlated protein profiles between compared lots. The color scale is based on the Pearson correlation coefficient’s *r* from 0.6 to 1; (B) Quantitative ELISA data for PEDF and FN1 across 12 independent UCMSC-EV production lots are shown; (C) Pathway and function distribution of the top 10% highly expressed proteins shared among three lots is shown. The pathway categories include cell communication, cell homeostasis, inflammatory related signaling, oxidative stress, RNA modulation, wound healing and ECM reorganization; (D) The dot plot visualizes the results of the pathway analysis on the top 10% protein expression data. The y-axis lists the significantly enriched biological pathways identified by the Qiagen IPA analysis (*P* < 0.05). The x-axis is the ratio, offering additional context by indicating the proportion of proteins from the experimental dataset that are included within a given pathway or disease/function category relative to the total number of molecules associated with that category in the IPA database. The color scale (blue to red) indicates the -log(P) value of that pathway whereas the size of each dot corresponds to the number of proteins from the experiment that are part of that specific pathway or category. Larger dots indicate a greater overlap between the identified proteins and the molecules within that pathway. DED: Dry eye disease; ECM: extracellular matrix; FN1: Fibronectin; PEDF: pigment epithelium-derived factor; IPA: Ingenuity Pathway Analysis; UCMSC-EVs: umbilical cord mesenchymal stem cell-derived extracellular vesicles; ELISA: enzyme-linked immunosorbent assay.

Proteomic analysis of UCMSC-EVs identified ~1,900 proteins, with the top 10% highly expressed proteins shared among three independent lots detailed in Supplementary Table 1. While literature often cites the heterogeneity of EV protein profiles as a primary hurdle in developing ophthalmic therapeutics^[7]^, our large-scale UCMSC-EV manufacturing process demonstrated consistency as shown by Pearson’s correlation analysis of the protein expression profile across three different lots. Label-free quantification was used to estimate and normalize protein abundances. Proteins with undetectable expression were excluded, resulting in a dataset containing 1,380 quantified proteins among all three samples evaluated here. The correlation plot was generated based on normalized protein expression levels derived from protein mass spectrometry data. The resulting heatmap revealed a high degree of correlation among these independent UCMSC-EV production lots with the *r* greater than 0.8 (*P* < 0.05) [Figure 2A].

Notably, several key immunomodulatory and regenerative proteins were consistently enriched across all evaluated lots. These include immunomodulatory proteins, such as CFH, SERPINF1 (PEDF), SOD1, and insulin-like growth factor-binding protein-5 (IGFBP-5), as well as proteins critical for extracellular matrix (ECM) remodeling and wound healing, such as FN1, TIMP1, COL1A2, and SPARC^[17]^. Among these, PEDF was prioritized for further investigation due to its established therapeutic role in mitigating DED-associated inflammation^[18-21]^. To bridge the proteomic findings with quantitative validation, we evaluated the expression of PEDF and FN1 across 12 independent UCMSC-EV lots using ELISA. The results confirmed a consistent protein load across all lots [Figure 2B], validating the robustness of the manufacturing process and supporting the potential for reproducible clinical efficacy.

IPA on these top 10% expressed proteins revealed associations with approximately 40 signaling pathways relevant to DED pathogenesis. These pathways were categorized into key biological processes, including immune modulation, mitigation of oxidative stress, promotion of wound healing, extracellular matrix reorganization, maintenance of cell homeostasis, and RNA modification [Figure 2C]. Notably, ~50% of the signaling pathways identified are related to oxidative stress and immunomodulation, while 28% of the signalings are related to wound healing. These findings suggest that the top expressed proteins in the UCMSC-EV proteome are functionally dedicated to modulating the drivers of DED progression.

Detailed dot plot analysis further demonstrated that UCMSC-EV proteins are strongly enriched in pathways essential for healing and tissue regeneration, such as regulation of IGF transport and uptake by IGFBP, integrin cell surface interaction, extracellular matrix organization, and collagen biosynthesis [Figure 2D]. Additionally, the proteome showed robust associations with core immunomodulatory signalings, including IL-6/JAK/STAT signaling, complement cascades, S100 family signaling, and transforming growth factor beta (TGF-β) signaling, which are critical for alleviating ocular surface inflammation in DED. Beyond these primary functions, the UCMSC-EV proteins were also associated with the binding and uptake of ligands by scavenger receptors as well as nuclear factor erythroid 2-related factor 2 (NRF2)-mediated oxidative stress response, indicating a multifaceted role in managing oxidative damage in DED [Figure 2D]. Intriguingly, IPA analysis identified a profound enrichment of proteins associated with the post-translational protein phosphorylation pathway [-log(p) = 22; Supplementary Table 2]. The presence of key regulatory proteins within this pathway, such as FN1 and SERPINA1, suggests a potential mechanism to modulate the PI3K/AKT and STAT signaling pathways, critical for epithelial wound healing and immunomodulation.

### UCMSC-EVs exhibit distinct miRNA profiles enriched with immunomodulatory regulators

**Figure 3:**
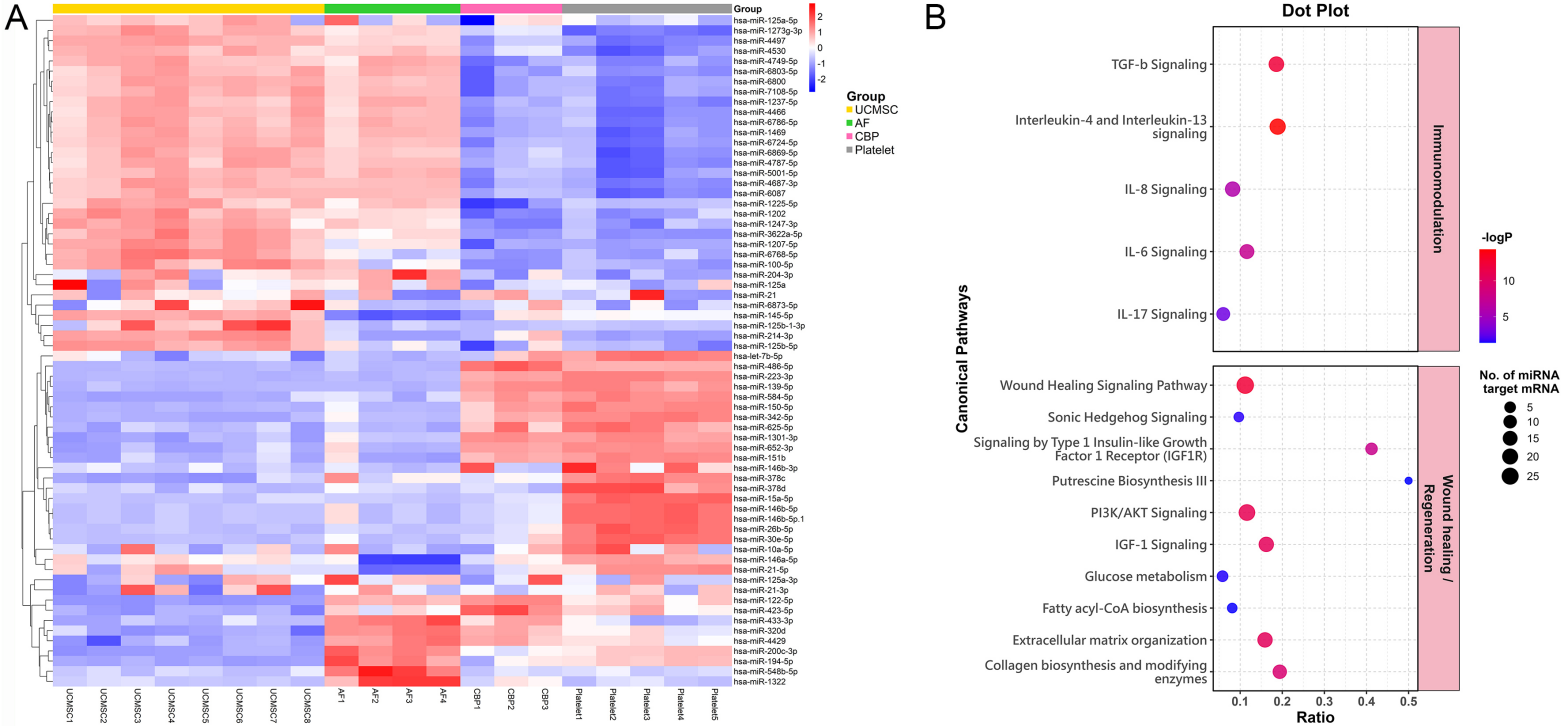
UCMSC-EVs have distinct miRNA profiles targeting DED-relevant mRNA pathways. (A) The heatmap analysis shows that UCMSC-EVs have distinct miRNA profiles as compared to AF, CBP, and platelet-derived EV samples; (B) The dot plot displays key pathways regulated by the miRNAs through their target mRNAs. The y-axis lists the significantly enriched biological pathways identified by the Qiagen IPA (*P* < 0.05). The x-axis is the ratio, offering additional context by indicating the proportion of miRNA-targeted RNAs from the experimental dataset that are included within a given pathway or disease/function category relative to the total number of mRNA molecules associated with that category in the IPA database. The color scale (blue to red) indicates the -log(P) value, whereas the size of each dot corresponds to the number of miRNAs whose target mRNAs are involved in that specific pathway or category. Larger dots indicate a greater overlap between the identified miRNAs and their targeted mRNAs. AF: Amniotic fluid; CBP: cord blood plasma; DED: dry eye disease; EV: extracellular vesicles; miRNA: microRNA; mRNA: messenger RNA; UCMSC-EVs: umbilical cord mesenchymal stem cell-derived extracellular vesicles; IPA: Ingenuity Pathway Analysis.

For clinical application of MSC-EVs, one regulatory consideration is to demonstrate the unique identity of the produced EVs compared with other sources, particularly for treating a specific disease of interest^[7]^. The literature has previously investigated the protein differences of UCMSC-EVs and those derived from bone marrow, adipose tissue and placenta^[22-24]^. These studies showed these EVs shared core therapeutic functions but their secretomes possess distinct, protein profiles. However, few studies have explored the differences among various EV types based on their miRNA profiles. To delineate unique miRNA cargoes, miRNA microarray analysis was performed on UCMSC-EVs and EVs derived from three other sources, including AF, CBP, and platelets. Figure 3A highlights the 25 most upregulated and 25 most downregulated miRNAs identified in the UCMSC-EVs as compared to AF, CBP or platelet-derived EVs. Specific miRNAs described in the literature for alleviating DED symptoms are also included in the heatmap^[9,25-27]^. The fold change and the *P*-value results of these comparisons are listed in Supplementary Table 3.

The heatmap revealed that UCMSC-EVs possess a distinct miRNA profile compared to other EV types, suggesting cell-source-specific functions. Specifically, miR-204, miR-21, and miR-100 were expressed significantly higher in UCMSC-EVs than in EVs from other sources. The biological relevance of these findings is supported by established mechanistic studies. For example, Zhou and coworkers showed that miR-204 from UCMSC-EVs targets the IL-6/IL-6R/Stat3 pathway, reprogramming pro-inflammatory M1 macrophage toward an immunosuppressive M2 phenotype, thus alleviating Graft-versus-Host Disease (GVHD)-associated DED^[27]^. Kim and coworkers validated the function of miR-21-5p by co-treating induced pluripotent stem cell (iPSC)-MSC EVs with miR-21 inhibitors, demonstrating that the inhibitory effects of EVs on the secretion of IL-6, IFN-r, and IL-17 were abrogated^[26]^. Similarly, using a miR-100-5p inhibitor, Li and coworkers demonstrated that miR-100-5p from UCMSC-EVs promoted macrophage polarization into M2 anti-inflammatory phenotype and increased T-regulatory cell expansion^[28]^. Together, these literature data suggest that the differentially expressed specific miRNAs identified in the UCMSC-EV miRNA microarray may exert therapeutic potential for DED.

Beyond these differentially expressed miRNAs, Supplementary Table 4 lists the top 10% highly expressed miRNAs in the UCMSC-EVs. Among them, let-7b was highly expressed across all eight UCMSC-EVs lots and has been found to mitigate DED via downregulating IRAK1/TAB2/NF-κB signaling^[29]^. Though not yet established for DED, another immunomodulatory miRNA, miR-6089, was also found to be highly expressed across all eight UCMSC-EVs lots. miR-6089 has been shown to exert anti-inflammatory function by targeting TLR4/NF-κB pathway in rheumatoid arthritis^[30]^. Both miRNAs target mRNAs that modulate the NFκB signaling, a critical transcription factor affecting many inflammatory related genes.

Furthermore, IPA analysis of the UCMSC-EV miRNA microarray data revealed that 334 out of 450 distinct miRNAs with expression levels above the median in UCMSC-EVs showed significant associations (*P* < 0.05) with specific signal transduction pathways. These include pathways crucial to UCMSC-EV function in DED, especially the immunomodulatory signaling such as IL-6/IL-8 signaling and TGF-β signaling as discussed in literatures^[26,27,30]^. The UCMSC-EVs miRNAs also strongly associate with many canonical pathways crucial for cell proliferation, regeneration, and wound healing, such as extracellular matrix organization, wound healing signaling pathway, IGF-1 signaling, collagen biosynthesis, PI3K/AKT signaling and sonic hedgehog signaling [Figure 3B].

By integrating these source-specific miRNA patterns with robust proteomic data, we provide a transparent molecular 'fingerprint' of our UCMSC-EVs that supports the presence of critical miRNAs and proteins with predicted immunomodulatory and tissue regenerative effects. This dual-omic characterization addresses the critical need for well-characterized identity profiles in the development of standardized EV-based therapeutics for DED.

### UCMSC-EVs suppress inflammatory responses in the human immune cell models

The intrinsic complexity of DED inflammation suggests that multi-modal therapies hold significant promise^[31]^. To move beyond molecular predictions, we sought to functionally validate the identified immunomodulatory and regenerative components through targeted *in vitro* assays. Consistent with the established therapeutic potential of MSC-EVs in alleviating inflammatory eye diseases^[7]^, the proteomic and miRNA profiling of UCMSC-EVs identified numerous components strongly associated with immune regulation and tissue regeneration. Building upon these molecular insights, we utilized two DED-relevant *in vitro* cell-based assays - wound healing and inflammation - to functionally validate the predicted therapeutic potential of the UCMSC-EVs.

**Figure 4:**
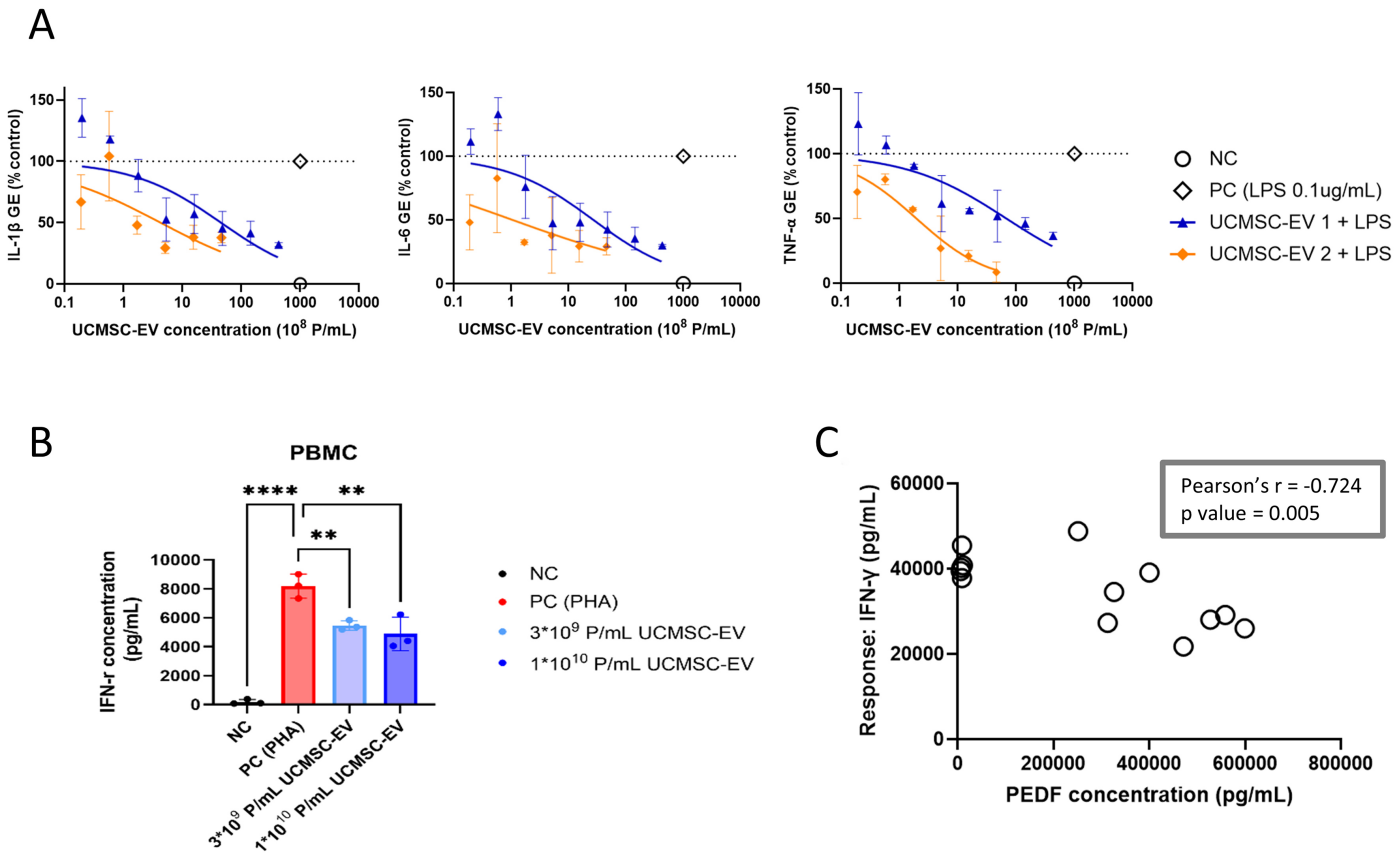
UCMSC-EVs suppress inflammatory cytokines in both LPS-induced THP-1 and PHA-induced PBMC inflammatory models. (A) The gene expression of inflammatory cytokines (IL-1β, IL-6, TNF-α) was quantified by RT-qPCR. The relative expression of each gene was first quantified from fold change (2^-ΔΔCt^) and then normalized in between LPS control (PC: 100%) and naïve state (NC: 0%). The dose-response curve shows percent cytokine gene expression at different UCMSC-EV concentrations compared to maximum cytokine levels (LPS alone without UCMSC-EVs; 100%). Data are presented as the mean ± SD, based on two independent biological replicates for the sample groups and three independent biological replicates for the control groups; (B) The bar graph shows that PHA markedly increased IFN-γ secretion in PBMCs (46-fold differences between positive and negative controls, ^****^P < 0.0001, one-way ANOVA with Dunnett’s post hoc test, *n* = 3 replicates). Treatment with UCMSC-EVs significantly reduced IFN-γ concentrations compared with PC groups (35%-42% reduction with EV treatment, ^**^P < 0.01, one-way ANOVA with Dunnett’s post hoc test, *n* = 3 replicates); (C) The dot plot showed the negative correlation between PEDF concentration of UCMSC-EVs quantified by ELISA and the IFN-γ concentrations measured from the UCMSC-EV treatments in the PHA-induced PBMC model. The *r* = -0.724, *P* = 0.005, *n* = 11 UCMSC-EV production lots. IL-1β: Interleukin-1 beta; IL-6: interleukin-6; IFN-γ: interferon gamma; LPS: lipopolysaccharide; NC: negative control; PBMCs: peripheral blood mononuclear cells; PC: positive control; PEDF: pigment epithelium-derived factor; PHA: phytohemagglutinin; RT-qPCR: reverse transcription quantitative polymerase chain reaction; THP-1: human monocytic leukemia cell line THP-1; TNF-α: tumor necrosis factor-alpha; UCMSC-EVs: umbilical cord mesenchymal stem cell-derived extracellular vesicles; SD: standard deviation; ANOVA: analysis of variance; ELISA: enzyme-linked immunosorbent assay.

To directly assess the anti-inflammatory function predicted by the molecular profiling, an LPS-induced monocytic THP-1 cell model was employed. The THP-1 cells were differentiated into M0 macrophages with 100 ng/mL PMA, followed by inflammatory response induction with 0.1 µg/mL LPS. To characterize the therapeutic window, a dose-response study was performed using eight different concentrations of UCMSC-EVs. Treatment with UCMSC-EVs attenuated the LPS-induced secretion of pro-inflammatory cytokines in the THP-1 cell model in a dose-dependent manner [Figure 4A]. Specifically, the concentration required to attenuate ~50% response of the TNF-α, IL-6, and IL-1β GE was approximately 10 to 100 × 10^8^ particles/mL.

These descriptive trends in GE were confirmed in the PHA-induced PBMC IFN-γ secretion model (*n* = 3), providing cytokine protein data [Figure 4B]. Treatment with UCMSC-EVs significantly reduced PHA-induced IFN-γ secretion (35%-42% reduction, *P* < 0.01, one-way ANOVA with Dunnett’s post hoc test, *n* = 3 replicates). Furthermore, a murine RAW264.7 model was established as a routine QC assay. UCMSC-EVs effectively reduced LPS-induced mIL-6 gene activation and protein secretion in this model [Supplementary Figure 1]. Analysis of twelve independent UCMSC-EV production lots showed consistent inhibition of IL-6 protein secretion, further validating the robustness of the manufacturing process [Supplementary Figure 1].

To bridge the gap between the proteomic predictions and functional outcomes, we performed a correlation study between a specific cargo candidate and the observed *in vitro* anti-inflammatory bioactivity. As discussed earlier, PEDF has an established therapeutic role in mitigating DED-associated inflammation^[18-21]^. Specifically, a PEDF-derived short peptide can relieve inflammatory response in rodent DED model and suppress MMP9 in a rabbit corneal epithelial cell model^[21]^. Supporting this, we observed a significant negative correlation between PEDF concentration, as quantified by ELISA, and IFN-γ secretion in the PHA-induced PBMC model (*r* = -0.724, *P* value = 0.005, *n* = 11 UCMSC-EV lots) [Figure 4C]. These data demonstrate that higher PEDF concentrations are associated with lower IFN-γ secretion, indicating enhanced anti-inflammatory potency. While we did not perform targeted knock-in or knock-out experiments to definitively validate this MoA, this correlation supports the role of PEDF as one of the potential molecular drivers for UCMSC-EV potency. Accordingly, these results demonstrate that UCMSC-EVs possess robust, lot-to-lot consistent anti-inflammatory activity that is quantitatively linked to their specific protein cargo, establishing a preliminary mechanistic basis for their therapeutic potential in DED.

### UCMSC-EVs promote wound healing in the HCE-S cell model

Impaired corneal wound healing and epithelial barrier disruption are hallmark clinical features of DED. Clinical diagnosis often relies on ocular surface staining, where a corneal fluorescein score > 5, conjunctival lissamine green staining > 9, or significant lid margin staining indicates pathological epithelial damage^[32]^. Established evidence showed that EVs from bone marrow-derived MSCs, corneal stromal stem cells, or adipose tissue-derived MSCs facilitated wound healing or promoted proliferation and migration of human cornea epithelial cells (HCEs)^[7]^. Here, we sought to examine the ability of UCMSC-EVs to promote ocular surface regeneration. A standardized scratch assay was performed on a confluent monolayer of HCE-S cells.

**Figure 5:**
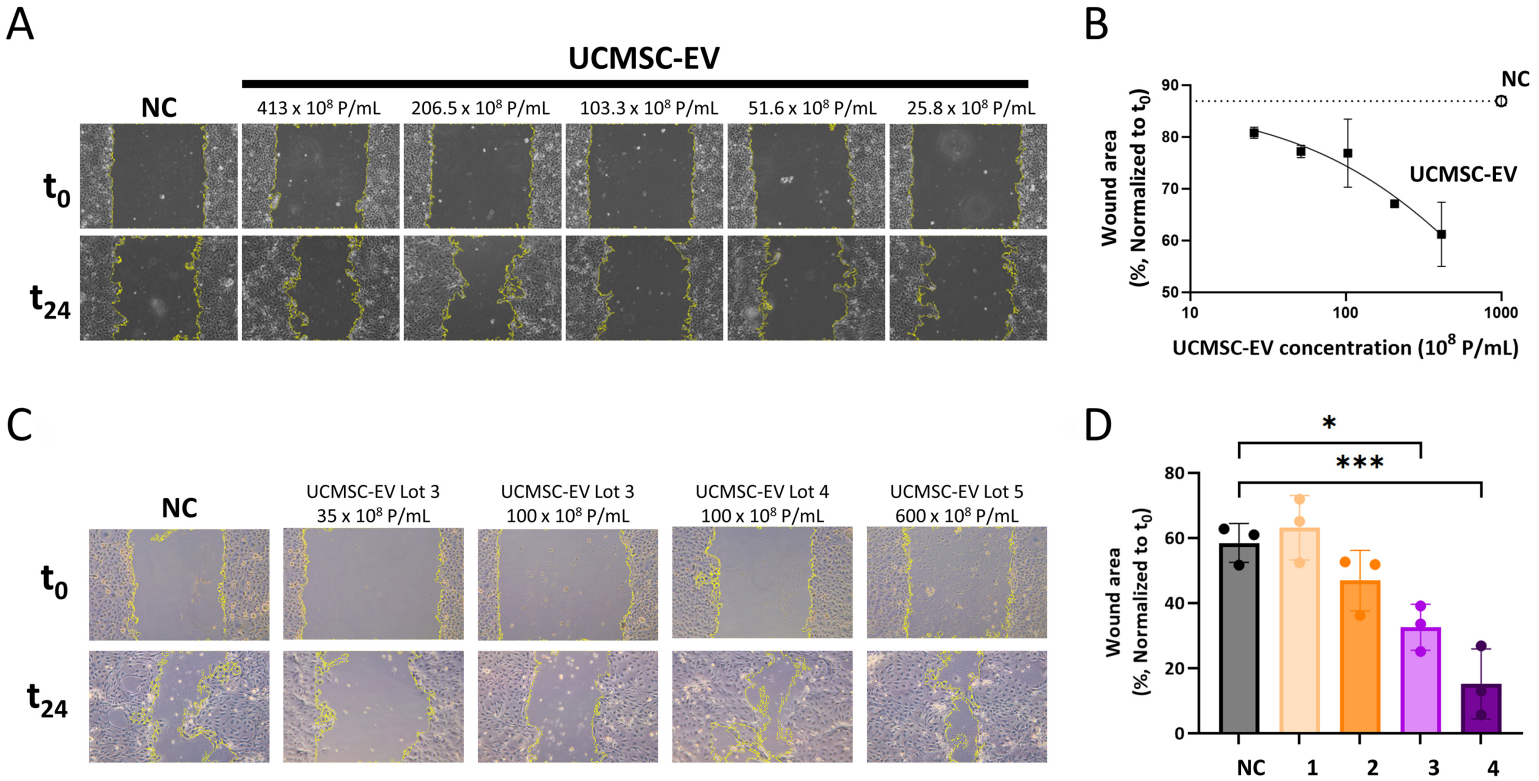
UCMSC-EVs promote wound healing of the human corneal epithelial cells in a dose dependent manner. (A) Bright field microscopic images show increased cell migration toward the empty area from UCMSC-EV-dosed wells after 24-h treatment; (B) Quantitative data from (A) show dose-dependent reduction of wound closure area as compared to the negative control (open circle). Data are presented as the mean ± SD, based on two independent biological replicates of each condition; (C) Bright- field microscopic images show increased cell migration across different lots of UCMSC-EV-dosed wells after 24-h treatment; (D) Quantitative data from (C) show significant reduction of wound closure area from UCMSC-EV lot 4 (Bar 3) and lot 5 (Bar 4) treatments as compared to the NC (black bar) (UCMSC-EV lot 4 100 × 10^8^ P/mL *vs*. NC: ^*^P = 0.0155, *n* = 3. UCMSC-EV lot 5 600 × 10^8^ P/mL *vs*. NC: ^***^P = 0.0004, *n* = 3, one-way ANOVA with Dunnett’s post hoc test). Bar 1: UCMSC-EV lot 3 35 × 10^8^ P/mL; Bar 2: UCMSC-EV lot 3 100 × 10^8^ P/mL; Bar 3: UCMSC-EV lot 4 100 × 10^8^ P/mL; Bar 4: UCMSC-EV lot 5 600 × 10^8^ P/mL. NC: Negative control; UCMSC-EVs: umbilical cord mesenchymal stem cell-derived extracellular vesicles; SD: standard deviation.

Following the creation of a uniform scratch using a P1000 pipette tip, HCE-S cells were treated with varying concentrations of UCMSC-EVs, with serum-free medium serving as the negative control. Wound closure was captured via bright-field microscopy at 0 and 24 h post-treatment. The UCMSC-EVs promoted HCE-S wound healing in a dose-dependent manner [Figure 5A]. The highest concentration of EVs accelerated the migration and closure of the corneal epithelial wound (~40% closure) as compared to the control group (~10% closure) [Figure 5B]. The dose-dependent response trend was further strengthened via the wound healing experiment examining across multiple production lots [Figure 5C and D]. All UCMSC-EV lots enhanced HCE-S wound closure. Specifically, UCMSC-EV lot 4 at 100 × 10^8^ P/mL and UCMSC-EV lot 5 at 600 × 10^8^ P/mL showed significant reduction in wound area as compared to negative control (*P* = 0.0155 and *P* = 0.0004, respectively; *n* = 3, one-way ANOVA with Dunnett’s post hoc test). In summary, the consistent wound healing performance observed in this assay underscores the potential of UCMSC-EVs to repair corneal surface disruption, a key factor contributing to the severity of DED.

## DISCUSSION

The findings of this study support the clinical development of UCMSC-EVs as a novel, cell-free therapeutic for the DED. The physicochemical characterization confirms that the isolated particles are extracellular vesicles, as evidenced by the morphology via cryo-EM, size distribution via NTA, and the positive expression of exosomal markers CD9, CD63, and CD81, via flow cytometry. Crucially, the high lot-to-lot correlation observed in the proteomic profiles demonstrates that the stability of the large production process, a prerequisite for clinical translation. Additionally, the distinct miRNA cargo of UCMSC-EVs, when compared to EVs from other human biological sources, highlights the suitability of the umbilical cord source for DED. Finally, the convergence of the miRNA and proteomic data via IPA analysis identified shared immunomodulatory and regenerative pathways, such as IL-6 signaling and IGF-1 signaling, indicating potential synergistic effects of UCMSC-EV miRNAs and proteins to mitigate DED progression.

This potential of UCMSC-EV indicated by the dual-omic data is functionally realized in the *in vitro* potency data, which address the two fundamental goals of DED treatment: ocular surface repair and inflammation control. The wound healing function observed in the human HCE-S cell model highlights the regenerative capacity of UCMSC-EVs, likely mediated by the ECM proteins, such as collagens (e.g., COL1A1, COL1A2), fibronectin (i.e., FN1), tissue inhibitors of metalloproteinase (i.e., TIMP1/2), cytoskeleton proteins (e.g., VIM, ACTG1, VCL), matricellular protein (i.e., SPARC), and growth factors including TGF-β, identified in the proteomic analysis data [Supplementary Table 1]. Simultaneously, the robust inhibition of inflammatory responses across three different inflammatory models, i.e., LPS-induced THP-1 cells, LPS-induced RAW264.7 cells, PHA-induced PBMCs, confirms their immunomodulatory capabilities. While the current study focuses on *in vitro* characterization, the physiological relevance of these findings is supported by our recent *in vivo* study in a severe rat DED model using the same UCMSC-EV manufacturing process^[10]^. In this study, topical EV application significantly accelerated corneal wound healing as observed by corneal fluorescein staining (CFS) and suppressed pro-inflammatory markers (IL-1β and TNF-α) as quantified by RT-qPCR, validating the potency observed in the current *in vitro* HCE-S and THP-1 assays. Furthermore, the significant correlation between an identified molecular candidate, PEDF, and the observed *in vitro* anti-inflammatory bioactivity strengthened the identification of UCMSC-EV as a potent therapeutic for DED.

**Figure 6:**
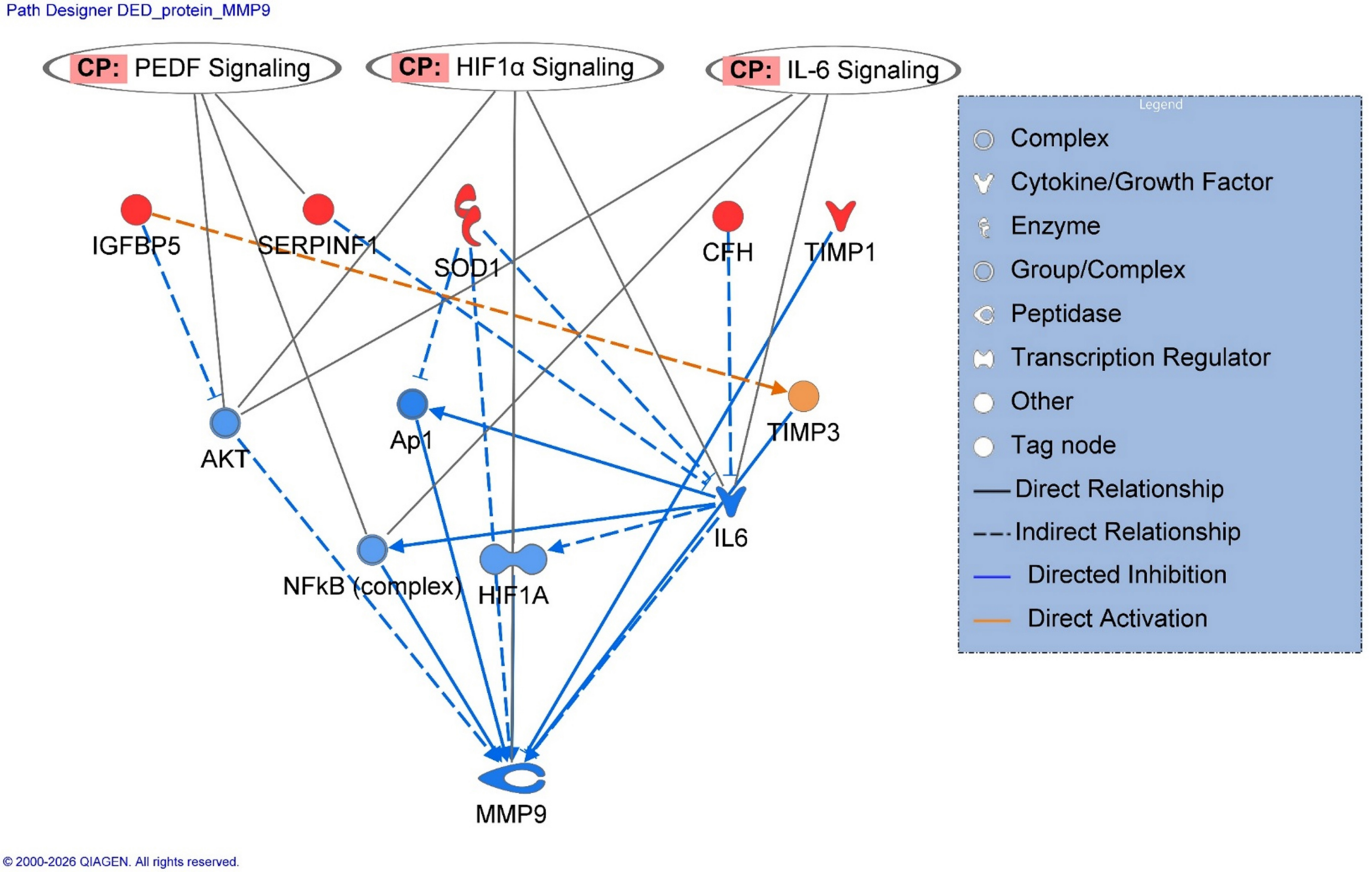
IPA-generated molecular interaction network illustrating the upstream and downstream regulatory relationships centered on the consistently highly expressed UCMSC-EV proteins, i.e., IGFBP5, PEDF/SERPINF1, SOD1, CFH, and TIMP1. The network shows that different upstream signaling converge on transcription factors, such as NFκB, HIF-1α, and AP-1, modulating the gene expression of MMP-9. The network was generated using QIAGEN IPA (QIAGEN Inc.)^[14]^. AKT: Protein kinase B; AP-1: activator protein 1; CFH: complement factor H; CP: canonical pathway; HIF-1α: hypoxia-inducible factor 1 alpha; IGFBP5: insulin-like growth factor binding protein 5; IL-6: interleukin-6; MMP-9: matrix metalloproteinase-9; NFκB: nuclear factor kappa B; PEDF: pigment epithelium-derived factor; SERPINF1: serpin family F member 1; SOD1: superoxide dismutase 1; TIMP1: tissue inhibitor of metalloproteinases 1; TIMP3: tissue inhibitor of metalloproteinases 3; IPA: Ingenuity Pathway Analysis; UCMSC-EVs: umbilical cord mesenchymal stem cell-derived extracellular vesicles.

Beyond broad functional effects, we sought to define the specific molecular drivers of this activity. Chronic inflammation in DED often leads to elevated levels of MMP-9, a protease that degrades the corneal epithelial basement membrane and tear film components, thereby perpetuating ocular surface damage^[12]^. Given its established correlations with disease severity, MMP-9 has become a critical diagnostic marker and a potential target for therapeutic intervention^[11,12]^. Through IPA-generated molecular interaction network analysis [Figure 6], we explored how the enriched protein cargo from the proteomic data, specifically PEDF (SERPINF1), TIMP1, CFH, SOD1, and IGFBP5, might influence the MMP-9 expression or/and activity. IPA analysis showed TIMP1 (tissue inhibitor of metalloproteinases-1) binds to and directly inhibits MMP-9^[33,34]^. The high MMP- to- TIMP1 ratio favors tissue degradation and inflammation^[35]^. IGFBP5 is predicted to modulate MMP-9 through activating TIMP3, another inhibitor of MMP-9, and reducing the AKT signaling based on IPA analysis. Literature data also showed IGFBP5 inhibits MMP9 via NFκB and HIF-1α signalings^[36,37]^. Finally, PEDF, SOD1, and CFH all inhibit MMP-9 via modulating IL-6 related signaling cascades, as predicted by IPA analysis. By integrating the proteomic data and the literature-based IPA analysis, we have established a robust molecular rationale for the multi-modal functions of UCMSC-EV against the chronic proteolytic and inflammatory environment of the DED ocular surface.

We acknowledge certain limitations in this study. First, the molecular interactions between UCMSC-EV cargo proteins and MMP-9 remain bioinformatics predictions generated through IPA analysis, and require further direct experimental validation. Second, while the internal controls define a clear therapeutic window, this study did not include a direct head-to-head benchmark against clinical standards of-care or other EV types; such comparative analyses remain a priority for future clinically relevant *in vivo* confirmatory studies. Finally, while the use of a single donor allowed us to isolate and confirm the robustness of the large-scale CMC manufacturing, the impact of donor-to-donor variability on these molecular profiles remains to be determined. Nevertheless, these data establish a directional mechanistic framework for the standardized manufacturing of UCMSC-EVs for future applications in DED.

In conclusion, this study provides a comprehensive molecular and functional characterization of UCMSC-EVs, establishing a robust foundation for their clinical development as a cell-free therapeutic for DED. By evaluating across multiple independent production lots, we demonstrated that UCMSC-EVs possess consistent, source-specific regenerative and anti-inflammatory properties that directly address the multifactorial pathogenesis of DED. The identification of PEDF, a key molecular driver and its correlation with functional potency, provides a directional mechanistic framework that bridges the gap between complex EV cargo and therapeutic efficacy. Although a significant amount of validation work remains to be performed, these findings pave the way toward ensuring the production of safe and consistent UCMSC-EVs as a next-generation ophthalmic therapy.

## DECLARATIONS

### Availability of data and materials

Data and materials will be provided upon availability and reasonable request.

### AI and AI-assisted tools statement

During the preparation of this manuscript, the AI tool Google Gemini (Large Language Model, accessed 2025-12) was used to assist in the literature search and to enhance manuscript readability and language quality. All cited references were subsequently verified by the authors for accuracy. Additionally, the Graphical Abstract was modified from an image generated using NotebookLM (developed by Google). The tool did not influence the study design, data collection, analysis, interpretation, or the scientific content of the work. All authors take full responsibility for the accuracy, integrity, and final content of the manuscript.

### Copyright

© The Author(s) 2026.
