# Supplementary material for: Molecular profiling and functional analyses of umbilical cord mesenchymal stem cell-derived extracellular vesicles for dry eye diseases
Source: Extracell Vesicles Circ Nucl Acids. 2026 Jun 16;7(2):927–44. doi: 10.20517/evcna.2025.177 (PMC13369709; doi:10.20517/evcna.2025.177)
Supplement: Supplementary file 1 [file evcna-7-2-927-SupplementaryMaterials.pdf]

Supplementary Materials

Molecular profiling and functional analyses of umbilical cord mesenchymal stem cell-derived extracellular vesicles for dry eye diseases

Qiao-Yu Hsu[1,#], Chia-Ni Hsiung[2,#], Hsin-Hung Cheng[3], Wen-Yu Lien[1], Wen-Hsien Lin[1,2], Martin Sieber[3], Chi-Chih Kang[1]

[1]BIONET Therapeutics Corp., Taipei 114065, Taiwan.

[2]Genetics Generation Advancement Corp. (GGA Corp.), Taipei 114065, Taiwan.

[3]BIONET Corp., Taipei 114065, Taiwan.

[#]These authors contributed equally to this work.

Correspondence to: Dr. Chi-Chih Kang, BIONET Therapeutics Corp., Taipei 114065, Taiwan. E-mail: ginnykang@bionettx.com

Supplementary Table 1. Top 10% highly expressed proteins identified in UCMSC-derived extracellular vesicles (UCMSC-EVs)

UniprotID	gene_name	Lot1	lot2	lot3	mean_abundance
P02768	ALB	4.12E+10	1.75E+10	1.32E+11	6.35E+10
P02751	FN1	1.11E+10	1.33E+10	5.55E+10	2.66E+10
P02452	COL1A1	1.01E+10	1.39E+10	2.42E+10	1.61E+10
P09486	SPARC	1.27E+10	1.13E+10	2.29E+10	1.56E+10
P01033	TIMP1	4.64E+09	1.3E+10	1.42E+10	1.06E+10
P08123	COL1A2	4.16E+09	4.37E+09	1.7E+10	8.52E+09
P21810	BGN	3.66E+09	4.66E+09	9.06E+09	5.79E+09
P08670	VIM	2.47E+09	3.34E+09	1.03E+10	5.37E+09
P63261	ACTG1	1.03E+09	1.97E+09	7.55E+09	3.52E+09
P12109	COL6A1	2.07E+09	1.43E+09	6.02E+09	3.17E+09
P13611	VCAN	8.46E+08	9E+08	7.65E+09	3.13E+09
Q16270	IGFBP7	1.93E+09	1.9E+09	4.96E+09	2.93E+09
P01024	C3	1.63E+09	1.02E+09	5.97E+09	2.87E+09
P01857	IGHG1	1.71E+09	7.53E+08	5.32E+09	2.59E+09
P02787	TF	1.43E+09	6.41E+08	5.01E+09	2.36E+09
P12111	COL6A3	1.19E+09	1.53E+09	4.19E+09	2.30E+09
P05121	SERPINE1	1.29E+09	1.77E+09	3.63E+09	2.23E+09
Q15582	TGFBI	9.13E+08	1E+09	4.54E+09	2.15E+09
P62937	PPIA	1.33E+09	1.75E+09	3.1E+09	2.06E+09
Q15063	POSTN	2.64E+08	4.15E+08	5.14E+09	1.94E+09
P08253	MMP2	1.07E+09	7.15E+08	4E+09	1.93E+09
Q12841	FSTL1	8.84E+08	1.22E+09	3.19E+09	1.77E+09
P21333	FLNA	7.73E+08	1.27E+09	2.86E+09	1.64E+09
P20908	COL5A1	9.01E+08	8.52E+08	3.15E+09	1.63E+09
P02461	COL3A1	7.26E+08	8.25E+08	2.99E+09	1.51E+09
O75369	FLNB	5.16E+08	7E+08	3.06E+09	1.43E+09
P35579	MYH9	4.83E+08	1.13E+09	2.27E+09	1.30E+09
P01023	A2M	7.52E+08	4.21E+08	2.61E+09	1.26E+09
P01009	SERPINA1	7.8E+08	3.07E+08	2.66E+09	1.25E+09

P06733	ENO1	6.13E+08	9.45E+08	2.11E+09	1.22E+09
P07996	THBS1	4.49E+08	6.02E+08	2.45E+09	1.17E+09
P00738	HP	8.33E+08	3.65E+08	2.26E+09	1.15E+09
Q01995	TAGLN	4.77E+08	7.26E+08	2.19E+09	1.13E+09
O43707	ACTN4	8.31E+08	5.77E+08	1.97E+09	1.13E+09
P02647	APOA1	2.8E+08	2.64E+08	2.68E+09	1.08E+09
P35555	FBN1	4.77E+08	4.93E+08	2.24E+09	1.07E+09
P51884	LUM	4.39E+08	6.61E+08	2.03E+09	1.04E+09
Q08629	SPOCK1	5.61E+08	7.21E+08	1.46E+09	9.14E+08
P12110	COL6A2	4.92E+08	4.59E+08	1.67E+09	8.74E+08
P18206	VCL	7.54E+08	1.29E+09	5.04E+08	8.49E+08
P07737	PFN1	5.08E+08	7.45E+08	1.22E+09	8.25E+08
P11142	HSPA8	5.67E+08	5.68E+08	1.32E+09	8.17E+08
O75326	SEMA7A	5.46E+08	6.31E+08	1.23E+09	8.03E+08
P60174	TPI1	5.54E+08	5.1E+08	1.34E+09	8.02E+08
P0DOX7	P0DOX7	5.68E+08	2.87E+08	1.54E+09	8.00E+08
Q99715	COL12A1	3.61E+08	1E+09	1.03E+09	7.97E+08
P08603	CFH	3.9E+08	3.38E+08	1.61E+09	7.81E+08
P00558	PGK1	5.64E+08	6.15E+08	1.15E+09	7.76E+08
P04075	ALDOA	5.3E+08	4.86E+08	1.22E+09	7.45E+08
P26038	MSN	4.47E+08	4.21E+08	1.28E+09	7.15E+08
P62328	TMSB4X	3.18E+08	7.41E+08	1.06E+09	7.07E+08
P07355	ANXA2	26886015	26064167	2.07E+09	7.06E+08
P09493	TPM1	5.19E+08	1.15E+09	3.32E+08	6.66E+08
P00338	LDHA	4.78E+08	5.13E+08	9.83E+08	6.58E+08
P67936	TPM4	1.86E+08	2.24E+08	1.53E+09	6.45E+08
P01859	IGHG2	4.09E+08	1.35E+08	1.34E+09	6.27E+08
P12814	ACTN1	5.13E+08	3.97E+08	9.69E+08	6.26E+08
P01876	IGHA1	3.43E+08	1.6E+08	1.37E+09	6.24E+08
P36955	SERPINF1	3.14E+08	2.15E+08	1.33E+09	6.20E+08
P07585	DCN	3.11E+08	5.62E+08	9.79E+08	6.17E+08
P19022	CDH2	4.16E+08	3.15E+08	1.11E+09	6.14E+08

Q9Y490	TLN1	1.64E+08	2.22E+08	1.45E+09	6.12E+08
Q15113	PCOLCE	3.94E+08	2.86E+08	1.15E+09	6.09E+08
P04406	GAPDH	93154303	1.9E+08	1.54E+09	6.08E+08
Q08380	LGALS3BP	3.46E+08	3.92E+08	1.01E+09	5.84E+08
P14618	PKM	1.2E+08	1.53E+08	1.38E+09	5.50E+08
Q00610	CLTC	2.31E+08	3.92E+08	9.91E+08	5.38E+08
P09936	UCHL1	2.65E+08	5.15E+08	7.29E+08	5.03E+08
P11021	HSPA5	3.4E+08	3.15E+08	7.61E+08	4.72E+08
P08572	COL4A2	45614949	31558900	1.31E+09	4.62E+08
P02790	HPX	2.38E+08	58920991	1.05E+09	4.48E+08
Q16610	ECM1	2.85E+08	2.46E+08	8.02E+08	4.44E+08
P0DOY2	IGLC2	3.01E+08	1.84E+08	8.36E+08	4.40E+08
P01871	IGHM	1.78E+08	1.73E+08	9.63E+08	4.38E+08
Q14766	LTBP1	1.43E+08	80626719	1.09E+09	4.37E+08
O00469	PLOD2	1.2E+08	1.25E+08	1.04E+09	4.28E+08
P63104	YWHAZ	1.74E+08	2.46E+08	8.48E+08	4.22E+08
P23284	PPIB	2.24E+08	3.24E+08	7.16E+08	4.21E+08
P09382	LGALS1	68557133	3.72E+08	7.92E+08	4.11E+08
P22392	NME2	2.82E+08	3.75E+08	5.44E+08	4.00E+08
Q15149	PLEC	1.84E+08	3.42E+08	6.3E+08	3.85E+08
P04264	KRT1	1.55E+08	43072725	9.34E+08	3.78E+08
P27797	CALR	1.31E+08	3.42E+08	6.56E+08	3.76E+08
P61769	B2M	1.93E+08	2.34E+08	6.73E+08	3.67E+08
Q16658	FSCN1	1.88E+08	2.43E+08	6.63E+08	3.65E+08
P07237	P4HB	1.46E+08	3.05E+08	6.23E+08	3.58E+08
Q9Y617	PSAT1	47370588	1.89E+08	8.36E+08	3.58E+08
Q02818	NUCB1	1.93E+08	2.07E+08	6.7E+08	3.57E+08
P08476	INHBA	1.24E+08	1.73E+08	7.55E+08	3.51E+08
P23528	CFL1	1.11E+08	87348946	8.44E+08	3.47E+08
O76061	STC2	1.58E+08	3.06E+08	5.22E+08	3.29E+08
O75083	WDR1	1.52E+08	2.83E+08	5.45E+08	3.27E+08
P06396	GSN	1.71E+08	2.4E+08	5.44E+08	3.18E+08

P30101	PDIA3	2.33E+08	2.18E+08	5.04E+08	3.18E+08
P16035	TIMP2	1.35E+08	1.19E+08	6.93E+08	3.16E+08
P02545	LMNA	1.22E+08	2.92E+08	5.24E+08	3.13E+08
P50395	GDI2	1.4E+08	1.95E+08	5.85E+08	3.07E+08
P06744	GPI	1.76E+08	2.27E+08	5.16E+08	3.06E+08
P40926	MDH2	2.13E+08	2.43E+08	4.52E+08	3.03E+08
P05997	COL5A2	1.59E+08	1.81E+08	5.68E+08	3.03E+08
P17948	FLT1	1.64E+08	1.78E+08	5.21E+08	2.88E+08
P63241	EIF5A	1.19E+08	1.62E+08	5.81E+08	2.87E+08
Q96CG8	CTHRC1	1.13E+08	2.74E+08	4.7E+08	2.86E+08
Q08431	MFGE8	2114272	2673146	8.44E+08	2.83E+08
P24593	IGFBP5	1.22E+08	3.44E+08	3.69E+08	2.79E+08
P29401	TKT	1.2E+08	1.68E+08	5.36E+08	2.75E+08
P98160	HSPG2	1.33E+08	1.67E+08	5.15E+08	2.72E+08
P04004	VTN	61527562	93589373	6.59E+08	2.71E+08
P37802	TAGLN2	96476528	2.28E+08	4.84E+08	2.69E+08
P68363	TUBA1B	38134636	52330407	7.17E+08	2.69E+08
P10599	TXN	1.66E+08	2.98E+08	3.43E+08	2.69E+08
Q9H299	SH3BGRL3	2.14E+08	1.94E+08	3.97E+08	2.68E+08
P11047	LAMC1	1.11E+08	1.22E+08	5.7E+08	2.68E+08
Q92626	PXDN	27876479	70763809	7.03E+08	2.67E+08
P07093	SERPINE2	53723218	1.61E+08	5.45E+08	2.53E+08
P63313	TMSB10	1.24E+08	2.57E+08	3.68E+08	2.49E+08
P0DP25	CALM3	1.9E+08	2.54E+08	2.89E+08	2.44E+08
P62258	YWHAE	2.67E+08	2.73E+08	1.83E+08	2.41E+08
P07195	LDHB	1.34E+08	1.84E+08	4.02E+08	2.40E+08
P10909	CLU	72241099	1.01E+08	5.43E+08	2.39E+08
P02462	COL4A1	1.02E+08	63034273	5.45E+08	2.37E+08
P13639	EEF2	40166692	65169751	5.93E+08	2.33E+08
Q13308	PTK7	1.43E+08	1.47E+08	4.07E+08	2.32E+08
P08133	ANXA6	4948910	8293248	6.8E+08	2.31E+08
O94985	CLSTN1	1.5E+08	1.3E+08	4.04E+08	2.28E+08

Q14112	NID2	79642009	1.63E+08	4.36E+08	2.26E+08
P62805	H4C4	1.25E+08	1.59E+08	3.93E+08	2.26E+08
P62805	H4C13	1.25E+08	1.59E+08	3.93E+08	2.26E+08
P62805	H4C6	1.25E+08	1.59E+08	3.93E+08	2.26E+08
P62805	H4C3	1.25E+08	1.59E+08	3.93E+08	2.26E+08
P62805	H4C8	1.25E+08	1.59E+08	3.93E+08	2.26E+08
P62805	H4C11	1.25E+08	1.59E+08	3.93E+08	2.26E+08
P62805	H4C5	1.25E+08	1.59E+08	3.93E+08	2.26E+08
P62805	H4C16	1.25E+08	1.59E+08	3.93E+08	2.26E+08
P62805	H4C9	1.25E+08	1.59E+08	3.93E+08	2.26E+08
P62805	H4C2	1.25E+08	1.59E+08	3.93E+08	2.26E+08
P62805	H4C12	1.25E+08	1.59E+08	3.93E+08	2.26E+08
P62805	H4C1	1.25E+08	1.59E+08	3.93E+08	2.26E+08
P62805	H4C15	1.25E+08	1.59E+08	3.93E+08	2.26E+08
P62805	H4C14	1.25E+08	1.59E+08	3.93E+08	2.26E+08
P35442	THBS2	65714805	1.49E+08	4.56E+08	2.24E+08
P07384	CAPN1	1406857	2047408	6.67E+08	2.23E+08
P07437	TUBB	15074883	21786974	6.26E+08	2.21E+08
P0C0L5	C4B	65122732	40070434	5.43E+08	2.16E+08
P0C0L5	C4B_2	65122732	40070434	5.43E+08	2.16E+08
O00391	QSOX1	75542691	98639870	4.54E+08	2.09E+08
P18669	PGAM1	1.49E+08	1.5E+08	3.16E+08	2.05E+08
P19823	ITIH2	31894776	33676105	5.48E+08	2.04E+08
Q01518	CAP1	98306567	1.13E+08	3.96E+08	2.03E+08
O75368	SH3BGRL	1.61E+08	1.16E+08	3.23E+08	2.00E+08
Q09666	AHNAK	1E+08	1.45E+08	3.55E+08	2.00E+08
P14174	MIF	2.34E+08	2.45E+08	1.16E+08	1.98E+08
Q14767	LTBP2	47477268	73193823	4.73E+08	1.98E+08
P50454	SERPINH1	69426610	76343237	4.42E+08	1.96E+08
P09211	GSTP1	79944754	1.25E+08	3.81E+08	1.95E+08
Q9BUD6	SPON2	79747003	93519542	4.1E+08	1.94E+08
P07858	CTSB	1.04E+08	1.58E+08	3.21E+08	1.94E+08

Q07954	LRP1	2.64E+08	1.68E+08	1.43E+08	1.92E+08
P07942	LAMB1	1.07E+08	1.31E+08	3.38E+08	1.92E+08
P00450	CP	45766635	45344992	4.81E+08	1.91E+08
P04083	ANXA1	5956936	4777710	5.56E+08	1.89E+08
P02774	GC	94467846	45688787	4.09E+08	1.83E+08
P08758	ANXA5	1493690	2926757	5.43E+08	1.82E+08
P68104	EEF1A1	27992451	37857362	4.8E+08	1.82E+08
Q06830	PRDX1	60320028	83746956	4.02E+08	1.82E+08
O43852	CALU	1.49E+08	1.14E+08	2.8E+08	1.81E+08
P02760	AMBP	87436444	84619017	3.68E+08	1.80E+08
P13797	PLS3	99961919	1.15E+08	3.23E+08	1.79E+08
P26022	PTX3	71250263	66979306	3.97E+08	1.78E+08
Q01469	FABP5	98480885	1.13E+08	3.23E+08	1.78E+08
Q9NRN5	OLFML3	24424005	62331095	4.42E+08	1.76E+08
P00441	SOD1	1.32E+08	1.27E+08	2.64E+08	1.74E+08
Q99497	PARK7	79562757	1.46E+08	2.93E+08	1.73E+08
P05067	APP	30953152	75040058	4.13E+08	1.73E+08
Q02809	PLOD1	84401107	73108436	3.51E+08	1.69E+08
P00736	P00736	74049197	87099257	3.43E+08	1.68E+08
P40925	MDH1	1E+08	1.28E+08	2.69E+08	1.66E+08
P00739	HPR	6826109	10251737	4.66E+08	1.61E+08
Q9UBP4	DKK3	1.22E+08	99363759	2.6E+08	1.61E+08
P02763	ORM1	81029608	43528784	3.52E+08	1.59E+08
P19652	ORM2	31980481	19712112	4.24E+08	1.58E+08
P09871	C1S	65484151	1.03E+08	3.06E+08	1.58E+08
P02652	APOA2	40913789	64508296	3.61E+08	1.55E+08
Q13219	PAPPA	1.01E+08	85095130	2.78E+08	1.55E+08
P30086	PEBP1	92587917	1.14E+08	2.53E+08	1.53E+08
P13693	TPT1	1.19E+08	82844647	2.55E+08	1.52E+08
P46777	RPL5	63082092	28161311	3.57E+08	1.49E+08
P10915	HAPLN1	46358666	49713441	3.49E+08	1.48E+08
P14543	NID1	90360231	78330618	2.73E+08	1.47E+08

P07602	PSAP	39993006	1.11E+08	2.78E+08	1.43E+08
P07900	HSP90AA1	9648185	23346929	3.86E+08	1.40E+08
P13929	ENO3	1828345	2489146	4.11E+08	1.38E+08
Q14019	COTL1	1.05E+08	1.89E+08	1.2E+08	1.38E+08
P60709	ACTB	83436549	1.09E+08	2.19E+08	1.37E+08
P04196	HRG	1.02E+08	61297237	2.41E+08	1.35E+08
P19827	ITIH1	58032474	34322545	3.03E+08	1.32E+08
P00491	PNP	1.31E+08	63724898	1.97E+08	1.30E+08
P78417	GSTO1	80851934	1.22E+08	1.86E+08	1.30E+08
O00299	CLIC1	44735198	62502669	2.75E+08	1.28E+08
O00468	AGRN	65749332	67574904	2.4E+08	1.24E+08
Q14315	FLNC	93092020	81539139	1.98E+08	1.24E+08
Q14203	DCTN1	2165160	2765167	3.64E+08	1.23E+08
P16070	CD44	93190062	87798164	1.87E+08	1.23E+08
P02679	FGG	43120791	54343203	2.65E+08	1.21E+08
P22692	IGFBP4	61471560	1.04E+08	1.93E+08	1.20E+08
P01861	IGHG4	79277261	25129119	2.54E+08	1.19E+08
P01042	KNG1	1.16E+08	18196878	2.22E+08	1.19E+08
Q9Y4K0	LOXL2	44199761	1.01E+08	2.1E+08	1.18E+08
Q13813	SPTAN1	69792676	70100256	2.04E+08	1.15E+08
P55287	CDH11	65234875	61952122	2.17E+08	1.15E+08
P08238	HSP90AB1	40863172	88630636	2.11E+08	1.13E+08
P00734	F2	39569103	15258368	2.81E+08	1.12E+08
O75874	IDH1	61841449	1.02E+08	1.7E+08	1.11E+08
P02654	APOC1	30897808	80884876	2.19E+08	1.10E+08
P01860	P01860	17973484	7810324	2.99E+08	1.08E+08

Protein expression levels are shown for three independent production lots (Lot1, Lot2, and Lot3). Accession numbers and corresponding gene symbols are provided. The FN1, TIMP1, PEDF/SERPINF1, CFH, SOD1, IGFBP5 were in the top 10% highly expressed protein list.

Supplementary Table 2. Top enriched canonical pathways identified by Ingenuity Pathway Analysis (IPA) based on top 10% highly expressed proteins

**Ingenuity canonical pathways**	**-log(p-value)**	**Ratio**	**Molecules**
Regulation of Insulin-like Growth Factor (IGF) transport and uptake by IGFBPs	23.2	0.153	ALB,APOA1,C3,CALU,CDH2,FBN1,FN1,FSTL1,IGFBP5,IGFBP7,LTBP1,MMP2,NUCB1,P4HB,SERPINA1,STC2,TF,TIMP1,VCAN
Post-translational protein phosphorylation	22.8	0.168	ALB,APOA1,C3,CALU,CDH2,FBN1,FN1,FSTL1,IGFBP5,IGFBP7,LTBP1,NUCB1,P4HB,SERPINA1,STC2,TF,TIMP1,VCAN
Extracellular matrix organization	19.4	0.15	BGN,COL1A1,COL1A2,COL3A1,COL5A1,COL5A2,COL6A1,COL6A2,COL6A3,DCN,FN1,HSPG2,LUM,SERPINE1,SPARC,VCAN
Integrin cell surface interactions	16	0.153	COL1A1,COL1A2,COL3A1,COL5A1,COL5A2,COL6A1,COL6A2,COL6A3,FBN1,FN1,HSPG2,LUM,THBS1
Collagen biosynthesis and modifying enzymes	15.6	0.179	COL12A1,COL1A1,COL1A2,COL3A1,COL5A1,COL5A2,COL6A1,COL6A2,COL6A3,P4HB,PCOLCE,PPIB
Binding and Uptake of Ligands by Scavenger Receptors	12.7	0.105	ALB,APOA1,CALR,COL1A1,COL1A2,COL3A1,HP,HPX,IGHA1,IGLC2,LRP1,SPARC

Syndecan interactions	12.5	0.296	ACTN1,COL1A1,COL1A2,COL3A1,COL5A1,COL5A2,FN1,THBS1
Signaling by PDGF	8.05	0.121	COL3A1,COL5A1,COL5A2,COL6A1,COL6A2,COL6A3,THBS1
Inhibition of Matrix Metalloproteases	7.7	0.158	A2M,HSPG2,LRP1,MMP2,TIMP1,TIMP2
ILK Signaling	7.63	0.0513	ACTG1,ACTN1,ACTN4,FLNA,FLNB,FN1,MYH9,TMSB4X,VCL,VIM
HIF1 alpha Signaling	7.4	0.0485	FLT1,GPI,HSPA5,HSPA8,LDHA,LDHB,MMP2,SERPINE1,TF,VIM
Interleukin-4 and Interleukin-13 signaling	7.34	0.0721	COL1A2,FN1,FSCN1,HSPA8,IGHG1,MMP2,TIMP1,VIM
Sheddase Signaling Pathway	6.54	0.0464	CDH2,COL1A1,COL1A2,FN1,IGFBP5,IGFBP7,MMP2,POSTN,TIMP1
LXR/RXR Activation	5.79	0.0569	ALB,APOA1,C3,HPX,SERPINA1,SERPINF1,TF
Leukocyte Extravasation Signaling	5.55	0.0421	ACTG1,ACTN1,ACTN4,MMP2,MSN,TIMP1,TIMP2,VCL
Gene and protein expression by JAK-STAT signaling after IL-12 stimulation	4.61	0.108	MIF,MSN,PPIA,SOD1
Complement cascade	3.41	0.0373	C3,CFH,IGHG1,IGHG2,IGLC2
IL-17A Signaling in Fibroblasts	3.29	0.05	COL1A1,COL1A2,FN1,P4HB

Detoxification of Reactive Oxygen Species	3.18	0.0811	P4HB,SOD1,TXN
S100 Family Signaling Pathway	3.15	0.0146	CALM3,IGHG1,IGHG2,IGHM,IGLC2,MMP2,MYH9,PDIA3,SERPINF1,TPM1,TPM4
IGF-1 Signaling	2.85	0.0381	IGFBP5,IGFBP7,YWHAE,YWHAZ
Signaling by TGFBR3	2.83	0.0612	INHBA,TIMP1,TIMP2
Sirtuin Signaling Pathway	1.97	0.0174	LDHA,LDHB,PGAM1,PGK1,SOD1
NRF2-mediated Oxidative Stress Response	1.76	0.0186	ACTG1,PPIB,SOD1,TXN
Role of JAK family kinases in IL-6-type Cytokine Signaling	1.31	0.026	SERPINA1,TIMP1

Pathways are ranked by −log(p-value) using a right-tailed Fisher's exact test. Ratio indicates the proportion of input molecules mapped to each pathway. Molecules contributing to each pathway are listed.

Supplementary Table 3. Differential expression analysis among UCMSC, CBP, AF, and platelet samples

Transcript.ID.Array.Design	UCMSC *vs.* CBP	UCMSC *vs.* AF	UCMSC *vs.* Platelet	AveExpr	*F*	*P* value	adj.*P*.Val
hsa-miR-100-5p	7.458635	3.375676	6.299775	5.38522	53.28924	2.51E-09	5.05E-08
hsa-miR-6768-5p	7.027713	4.462602	6.122622	4.512516	57.10411	1.41E-09	3.38E-08
hsa-miR-1225-5p	6.824154	0.751539	3.915023	7.113337	60.50717	8.69E-10	2.48E-08
hsa-miR-1207-5p	6.623284	2.607671	5.878207	8.704019	74.47651	1.49E-10	6.45E-09
hsa-miR-1247-3p	6.468568	1.387745	5.283879	5.658195	55.98712	1.66E-09	3.79E-08
hsa-miR-1202	6.189266	1.528467	4.738921	5.119197	62.22521	6.86E-10	2.12E-08
hsa-miR-4749-5p	5.925763	0.096142	5.396938	7.533739	67.78206	3.33E-10	1.23E-08
hsa-miR-125b-5p	5.624807	3.454556	5.719665	7.631099	21.20638	3.17E-06	1.53E-05
hsa-miR-3622a-5p	5.05	1.716917	4.984043	4.288943	88.86765	3.26E-11	1.65E-09
hsa-miR-145-5p	4.139391	9.764968	3.607538	7.775665	63.4601	5.82E-10	1.91E-08
hsa-miR-1273g-3p	4.091839	1.67395	8.127929	10.51848	165.7859	1.33E-13	1.72E-11
hsa-miR-214-3p	4.074785	7.747686	8.320572	5.045958	57.46268	1.34E-09	3.26E-08
hsa-miR-6800	4.058548	0.479845	3.862287	9.671637	69.8069	2.59E-10	1.01E-08
hsa-miR-7108-5p	3.995523	0.341566	3.865421	10.08957	64.37442	5.16E-10	1.73E-08
hsa-miR-4497	3.853941	1.116065	5.06881	11.15871	132.1526	1.01E-12	8.37E-11
hsa-miR-6724-5p	3.654015	0.473826	4.214816	10.39527	67.42714	3.48E-10	1.25E-08
hsa-miR-4687-3p	3.637446	0.217553	4.287806	10.37224	101.8619	9.95E-12	6.84E-10

hsa-miR-5001-5p	3.511509	0.005152	4.463515	9.212586	60.68279	8.48E-10	2.46E-08
hsa-miR-1469	3.429244	0.222777	4.096792	10.13088	72.61882	1.85E-10	7.71E-09
hsa-miR-6869-5p	3.285507	0.548734	4.653779	11.01652	55.91504	1.68E-09	3.79E-08
hsa-miR-6087	3.241465	0.044599	4.517861	11.79412	96.17042	1.64E-11	9.56E-10
hsa-miR-6803-5p	3.199345	0.057969	2.80747	10.36253	58.13023	1.22E-09	3.07E-08
hsa-miR-4466	3.07628	0.256569	3.636989	8.728428	61.80897	7.26E-10	2.20E-08
hsa-miR-6786-5p	3.027163	0.136898	3.269237	10.69234	59.57196	9.90E-10	2.74E-08
hsa-miR-4787-5p	3.024647	0.2425	4.364632	11.27455	55.43948	1.80E-09	4.01E-08
hsa-miR-1237-5p	2.956015	0.053407	3.52718	10.68218	58.55831	1.14E-09	2.98E-08
hsa-miR-125b-1-3p	2.622863	2.471402	2.553387	1.954203	10.36154	0.000313	0.000852
hsa-miR-4530	2.05545	0.329615	3.496405	6.848229	62.75782	6.39E-10	2.01E-08
hsa-miR-125a-5p	2.05256	0.687197	1.650689	7.300667	2.051552	0.14146	0.17111
hsa-miR-204-3p	1.14694	-1.49554	1.672317	2.823223	8.543132	0.000893	0.002168
hsa-miR-21-5p	0.953134	3.402059	-2.24186	4.585383	12.22408	0.000119	0.000359
hsa-miR-125a	0.56766	-0.0626	0.394228	1.186761	1.343426	0.290669	0.321972
hsa-miR-146a-5p	0.49926	5.501329	-2.56805	7.517919	19.22955	6.30E-06	2.71E-05
hsa-miR-6873-5p	0.364897	0.706208	0.789619	1.536098	2.213167	0.120543	0.150132
hsa-miR-21-3p	0.166493	-0.03493	-0.12463	1.693967	0.130498	0.940737	0.942608
hsa-miR-21	-0.0614	0.224955	0.10338	1.137635	0.618459	0.611742	0.63834

hsa-miR-378d	-0.08164	-0.2922	-2.56138	1.54657	46.20296	8.09E-09	1.12E-07
hsa-miR-548b-5p	-0.08725	-2.86495	-0.14602	1.450247	58.92273	1.09E-09	2.88E-08
hsa-miR-10a-5p	-0.17325	-0.00604	-1.05191	1.962953	1.254875	0.318554	0.349025
hsa-miR-15a-5p	-0.58984	-0.04226	-6.28856	2.599172	290.1995	8.51E-16	2.57E-13
hsa-miR-1322	-0.64253	-2.87734	-0.02415	1.61349	37.95473	3.94E-08	3.94E-07
hsa-miR-26b-5p	-0.67585	-0.1727	-3.58852	1.884323	131.4843	1.05E-12	8.37E-11
hsa-miR-125a-3p	-0.68917	-1.57491	-0.1843	2.826825	1.276681	0.311444	0.342226
hsa-miR-146b-3p	-0.77775	0.074894	-1.69831	1.455151	6.875995	0.002618	0.005583
hsa-miR-146b-5p	-0.81304	-0.58866	-6.6758	2.981333	96.44374	1.60E-11	9.56E-10
hsa-let-7b-5p	-1.43601	0.201312	-3.02026	10.28385	18.94073	6.99E-06	2.94E-05
hsa-miR-30e-5p	-1.52922	-0.34656	-5.78812	2.681435	91.91958	2.44E-11	1.27E-09
hsa-miR-378c	-1.6829	-2.48231	-4.86988	3.121928	37.57135	4.27E-08	4.15E-07
hsa-miR-320d	-2.39612	-3.13416	-1.35254	9.597108	55.93836	1.67E-09	3.79E-08
hsa-miR-625-5p	-2.9489	-0.02266	-3.71162	2.780175	44.53369	1.09E-08	1.43E-07
hsa-miR-194-5p	-3.06277	-9.15063	-6.00607	4.834692	60.75599	8.39E-10	2.46E-08
hsa-miR-423-5p	-3.40429	-1.48017	-1.83768	7.89466	46.92272	7.14E-09	1.05E-07
hsa-miR-4429	-3.49199	-4.23282	-2.25192	7.501298	48.7591	5.21E-09	8.38E-08
hsa-miR-200c-3p	-3.89336	-9.91854	-6.54521	5.286378	165.3941	1.36E-13	1.72E-11
hsa-miR-433-3p	-4.30674	-6.4799	-0.59496	3.810198	51.41149	3.37E-09	6.37E-08

hsa-miR-151b	-4.86032	-0.29866	-5.98584	3.936872	50.36112	4.00E-09	7.19E-08
hsa-miR-1301-3p	-4.94664	-1.20379	-4.86355	3.659679	100.5019	1.12E-11	7.05E-10
hsa-miR-342-5p	-5.18539	-1.18162	-7.19473	3.728803	148.6271	3.54E-13	3.57E-11
hsa-miR-139-5p	-5.94263	-0.00902	-5.86178	3.175426	268.7769	1.71E-15	3.69E-13
hsa-miR-652-3p	-6.62666	-1.09841	-7.3511	4.523179	54.92757	1.95E-09	4.15E-08
hsa-miR-584-5p	-6.85587	-1.29222	-7.74042	4.636254	133.118	9.43E-13	8.37E-11
hsa-miR-223-3p	-7.26567	-0.20394	-8.7206	4.14868	988.223	1.11E-20	1.67E-17
hsa-miR-150-5p	-8.32606	-1.01523	-10.5712	4.960637	152.0286	2.89E-13	3.12E-11
hsa-miR-486-5p	-10.6057	-0.26381	-7.22733	4.54662	542.2756	2.79E-18	2.11E-15
hsa-miR-122-5p	-11.8868	-9.63646	-7.51389	6.645254	238.4335	5.06E-15	8.50E-13

Log2 fold changes for UCMSC versus CBP (cord blood plasma), AF (amniotic fluid) , and platelet derived EV samples are shown together with average expression values (AveExpr), F-statistics, raw P-values, and Benjamini–Hochberg adjusted P-values (adj.P.Val). The positive value means higher miRNA expression in UCMSC-EVs than in EVs from other sources whereas the negative value means less miRNA expression in UCMSC-EVs than in EVs from other sources.

Supplementary Table 4. Top 10% highly expressed miRNAs across eight independent UCMSC-EV lots

Accession	Transcript.ID.Array.Design	Lot 1	Lot 2	Lot 3	Lot 4	Lot 5	Lot 6	Lot 7	Lot 8
MIMAT0000063	hsa-let-7b-5p	10.64593	10.11153	9.02597	8.117842	9.469599	9.605411	9.840334	8.012538
MIMAT0005871	hsa-miR-1207-5p	11.54311	11.43073	12.56061	12.53913	11.09261	12.27455	11.79592	10.27211
MIMAT0000421	hsa-miR-122-5p	0.954348	0.81525	0.769619	1.62777	0.499761	1.016112	1.241414	1.527514
MIMAT0022941	hsa-miR-1227-5p	9.436481	10.07778	10.37248	10.99953	10.68961	9.999469	9.396251	11.15464
MIMAT0005582	hsa-miR-1228-5p	10.83851	11.47086	11.63478	11.72731	11.28178	10.89853	10.73414	11.89429
MIMAT0022943	hsa-miR-1233-5p	7.204839	8.384688	7.964899	9.493409	8.380479	7.429231	7.066974	7.745445
MIMAT0022946	hsa-miR-1237-5p	11.3488	12.10738	12.52295	12.61733	11.70304	11.78906	11.5237	12.53219
MIMAT0005898	hsa-miR-1246	10.63007	10.86913	9.489198	11.21166	10.42624	6.549459	6.312412	10.4037
MIMAT0005922	hsa-miR-1268a	9.154343	9.156687	8.927	9.449253	9.824123	7.555019	7.399625	9.831596
MIMAT0018925	hsa-miR-1268b	8.985992	9.138011	8.767631	9.424692	9.781679	7.848489	7.237335	9.456052
MIMAT0022742	hsa-miR-1273g-3p	13.17848	13.387	14.77513	14.19902	12.75166	13.65484	13.73258	12.3135
MIMAT0000446	hsa-miR-127-3p	5.623239	5.333132	6.982653	4.60915	2.282896	7.810941	7.95489	4.093154
MIMAT0005929	hsa-miR-1275	8.65675	9.477133	9.199864	10.03506	9.354213	9.111029	8.774435	7.16238
MIMAT0027038	hsa-miR-1343-5p	9.749603	10.52891	10.33309	10.91331	10.27912	9.64115	9.238975	10.2387
MIMAT0007347	hsa-miR-1469	11.00043	11.59245	12.0668	12.31071	11.44279	11.89675	11.39295	12.00928
MIMAT0004609	hsa-miR-149-3p	11.77648	12.36679	12.70651	12.50448	11.72382	12.33904	11.88953	12.08889

MIMAT0004611	hsa-miR-185-3p	0.926613	2.052232	4.534486	2.018786	0.848558	2.357053	2.12917	2.018786
MIMAT0007881	hsa-miR-1908-5p	11.03209	11.99001	11.6932	12.31828	11.18793	11.38728	11.26303	12.33332
MIMAT0007883	hsa-miR-1909-3p	8.422125	9.411472	9.709866	10.7697	9.786571	8.548773	7.848481	10.15548
MIMAT0007892	hsa-miR-1915-3p	11.67403	12.26381	12.86969	12.87797	12.00944	12.81011	12.8502	12.79521
MIMAT0000440	hsa-miR-191-5p	9.253846	8.712877	9.967568	8.979083	9.253385	9.95274	10.34205	9.202686
MIMAT0000460	hsa-miR-194-5p	1.320512	0.92475	1.630361	0.784142	1.250093	0.636971	0.777319	1.024913
MIMAT0000617	hsa-miR-200c-3p	0.294881	0.890715	0.735022	0.966587	1.013767	1.859626	1.927885	0.970428
MIMAT0000078	hsa-miR-23a-3p	12.12661	11.77138	12.37799	12.04348	11.79708	11.96247	12.07747	11.79418
MIMAT0000080	hsa-miR-24-3p	12.53438	11.80132	11.8775	11.84063	12.46077	11.70881	12.29203	11.67684
MIMAT0013802	hsa-miR-2861	11.48535	11.83904	12.75374	12.77752	12.15115	12.59742	12.59353	12.93269
MIMAT0004450	hsa-miR-297	8.156145	9.54766	5.913522	5.344897	7.944586	2.063156	3.919442	2.594443
MIMAT0018985	hsa-miR-3135b	9.355837	9.697311	9.124128	7.76335	6.830185	7.306219	6.99299	9.978997
MIMAT0015010	hsa-miR-3141	9.201317	10.02176	10.23675	10.76184	10.52887	9.040237	8.528124	10.46493
MIMAT0015055	hsa-miR-3178	11.57942	12.26512	11.39143	11.46464	11.32034	10.93956	11.17209	12.24385
MIMAT0015065	hsa-miR-3185	9.577264	9.875495	10.84541	11.29473	10.90404	10.84802	10.09108	11.24505
MIMAT0015080	hsa-miR-3196	11.46055	12.29788	12.84393	12.9207	11.55197	12.60045	12.67804	12.50111
MIMAT0000510	hsa-miR-320a	10.44965	10.54837	9.749073	9.726148	10.68184	10.63461	10.88927	8.990842
MIMAT0005792	hsa-miR-320b	10.16317	10.31602	9.648825	9.584322	10.74923	10.51573	10.789	8.870085
MIMAT0005793	hsa-miR-320c	10.23169	10.13533	9.623394	9.580369	10.69519	10.43677	10.70532	8.881166

MIMAT0006764	hsa-miR-320d	9.127861	8.3178	7.9694	8.119838	8.316363	8.414716	8.477859	7.437941
MI0014234	hsa-miR-320e	9.575232	10.22774	5.245751	5.963964	9.68115	7.622145	6.195587	7.101658
MIMAT0015072	hsa-miR-320e	4.583788	2.741778	4.987146	5.30095	3.600046	4.26755	5.094364	4.855791
MIMAT0026486	hsa-miR-328-5p	10.18572	10.87248	10.84798	11.39822	10.97042	10.36576	9.701491	11.09504
MIMAT0022967	hsa-miR-3620-5p	7.650237	8.721891	9.123605	9.585205	9.273713	8.680485	8.316788	9.416147
MIMAT0018002	hsa-miR-3621	7.840134	9.688377	9.422969	10.52153	9.810277	8.619889	8.230743	9.943265
MIMAT0018076	hsa-miR-3656	12.18166	12.4232	13.36211	13.61529	13.22821	12.64374	12.621	14.0035
MIMAT0018087	hsa-miR-3665	12.38724	12.696	14.07394	14.33407	12.91505	14.03194	13.94386	13.40834
MIMAT0019229	hsa-miR-3940-5p	11.15753	11.58473	12.59764	12.5541	11.82533	12.19756	11.76371	12.42753
MIMAT0019337	hsa-miR-3960	12.80493	12.94983	14.47576	15.06058	13.01526	14.00068	14.11311	13.92673
MIMAT0016900	hsa-miR-4270	9.322405	9.892929	10.48978	10.72803	10.48346	10.30391	9.523958	10.30952
MI0015885	hsa-miR-4281	8.607424	8.798334	9.091205	9.905221	9.460307	9.253813	8.548861	9.313173
MIMAT0016907	hsa-miR-4281	10.25179	10.90267	11.09445	11.31994	11.17804	10.51489	10.3552	11.11481
MIMAT0018944	hsa-miR-4429	5.315568	3.94654	5.910515	6.46093	5.426029	5.988505	6.033152	5.462394
MIMAT0018949	hsa-miR-4433-3p	6.352379	7.873103	8.196299	8.935774	8.517697	7.353745	7.249665	9.165385
MIMAT0030414	hsa-miR-4433b-3p	9.286279	9.972611	10.12042	10.5971	10.38335	9.314787	8.751627	10.67819
MIMAT0018958	hsa-miR-4440	3.540021	4.655232	6.726895	4.363999	4.530833	5.783205	6.042982	2.232499
MIMAT0018961	hsa-miR-4443	0.564287	2.182078	5.550402	4.526802	2.031402	5.182907	4.671361	5.66729
MIMAT0018976	hsa-miR-4454	9.913352	10.19277	11.71924	12.28306	10.15367	11.98354	11.90939	8.494501

MIMAT0018981	hsa-miR-4459	9.531637	9.519899	9.352814	9.940227	9.665365	7.685753	7.257838	8.369329
MIMAT0018986	hsa-miR-4462	2.578558	4.751493	5.930729	5.79319	4.295091	4.226929	4.372372	4.131544
MIMAT0018987	hsa-miR-4463	9.749603	10.46266	10.80398	11.10505	10.98698	10.48969	10.20186	11.22813
MIMAT0018993	hsa-miR-4466	11.9111	12.51694	13.17458	13.17625	12.82563	12.9253	12.96106	13.28054
MIMAT0018994	hsa-miR-4467	9.412025	10.23746	10.26031	11.28706	9.610535	9.523059	9.344265	11.06912
MIMAT0019018	hsa-miR-4484	12.92866	13.11454	12.44535	12.71839	12.69837	10.48614	9.793179	13.79299
MIMAT0019020	hsa-miR-4486	6.058364	5.700567	7.842764	8.619368	7.913054	7.846462	6.941923	9.782401
MIMAT0019022	hsa-miR-4488	11.96174	12.46836	13.15459	13.30988	12.67885	12.67074	12.69721	12.61359
MIMAT0019027	hsa-miR-4492	8.258939	10.28778	9.249921	11.44226	8.847139	8.899208	8.830331	9.348729
MIMAT0019032	hsa-miR-4497	13.2887	13.21822	13.4891	13.78064	13.27635	12.86355	12.94803	12.95317
MIMAT0019038	hsa-miR-4502	7.32226	7.663189	4.391527	3.789387	3.608223	1.25686	1.517756	1.776292
MIMAT0019041	hsa-miR-4505	7.503443	8.656032	8.579515	9.259435	8.957136	8.205564	7.708923	9.702569
MIMAT0019044	hsa-miR-4507	7.238854	7.146163	7.75477	8.710262	8.786513	8.316571	7.595582	8.43954
MIMAT0019045	hsa-miR-4508	11.26296	12.00669	12.13607	13.04688	11.35503	12.09391	11.76759	11.51083
MIMAT0019053	hsa-miR-4516	11.59402	12.194	12.991	13.0385	12.08343	12.73587	12.74537	12.68647
MIMAT0019069	hsa-miR-4530	11.69969	12.24419	12.6521	12.54174	11.20848	12.57891	12.6464	11.30106
MIMAT0019071	hsa-miR-4532	10.01592	10.65645	10.50966	11.33601	10.6407	9.857511	9.547951	11.24966
MIMAT0022977	hsa-miR-4632-5p	9.000593	8.757519	9.177756	9.448363	9.062075	8.797334	7.815735	9.104007
MIMAT0019711	hsa-miR-4649-5p	7.658694	8.94699	9.337917	10.32049	9.65063	8.264835	7.66307	10.14992

MIMAT0019715	hsa-miR-4651	8.807598	9.487732	10.6011	10.93713	10.69048	10.24266	9.519395	10.64132
MIMAT0019756	hsa-miR-4674	7.079249	8.373835	9.171943	10.06316	8.683303	9.2074	8.291613	9.371396
MIMAT0019775	hsa-miR-4687-3p	11.47742	11.59641	12.74745	12.59855	11.94555	12.3144	11.86799	11.71881
MIMAT0019778	hsa-miR-4689	8.608789	8.918719	10.21727	10.35458	9.932905	9.747197	9.127364	9.788513
MIMAT0019788	hsa-miR-4695-5p	8.109319	8.482955	9.140479	10.2348	9.174654	9.056982	8.033694	10.14973
MIMAT0019807	hsa-miR-4707-5p	10.59653	10.62086	11.40099	11.61936	11.25297	11.20223	10.69832	12.16852
MIMAT0019859	hsa-miR-4734	10.05528	10.37803	11.50344	11.60005	11.19894	11.33407	10.85052	12.02453
MIMAT0019868	hsa-miR-4739	9.795743	10.47907	10.71281	10.93582	9.752536	10.10267	9.403401	10.00711
MIMAT0019871	hsa-miR-4741	9.188654	9.683963	10.67443	10.96599	10.31099	10.34468	9.529544	10.52971
MIMAT0019878	hsa-miR-4745-5p	10.90337	10.96044	11.21574	11.34595	10.98333	11.14052	10.78779	11.32816
MIMAT0019885	hsa-miR-4749-5p	9.537619	10.27985	10.45433	10.89442	9.415859	9.341748	9.428146	8.976551
MIMAT0019903	hsa-miR-4758-5p	8.563226	9.384312	9.413631	10.06171	9.820346	9.329382	8.825185	9.960372
MIMAT0019913	hsa-miR-4763-3p	10.60202	10.66897	11.70524	11.567	11.40756	11.45028	11.20789	11.47759
MIMAT0019956	hsa-miR-4787-5p	11.85894	12.55368	13.29502	13.41869	12.17203	13.51496	13.385	12.74489
MIMAT0019966	hsa-miR-4793-3p	5.054443	3.326199	6.036625	3.280334	3.682429	5.507422	5.741592	1.63314
MIMAT0004761	hsa-miR-483-5p	5.216006	6.448108	5.431248	7.346711	6.285162	4.237003	2.920027	7.969903
MIMAT0021021	hsa-miR-5001-5p	10.19693	10.64991	10.73789	11.51541	10.70176	11.12436	10.751	11.1725
MIMAT0021085	hsa-miR-5093	1.329152	2.922893	5.472156	3.673919	1.539658	4.226929	4.320223	2.908398
MI0018001	hsa-miR-5095	8.003286	9.201688	8.619844	9.370712	8.230225	8.45852	8.112395	8.858327

MIMAT0022259	hsa-miR-5100	4.187017	6.910152	8.891178	9.052229	2.839808	8.900401	8.180065	3.405223
MIMAT0023252	hsa-miR-5787	11.57176	12.24065	12.60593	12.86342	12.06935	12.14856	11.72608	12.75329
MIMAT0023693	hsa-miR-6068	8.048075	8.141966	8.712399	10.34612	9.088218	9.449047	9.175502	9.619749
MIMAT0023710	hsa-miR-6085	9.033186	10.05333	9.532457	10.56889	10.05659	9.41441	8.75131	9.681528
MIMAT0023712	hsa-miR-6087	12.89271	13.06066	13.63664	14.19698	13.10918	13.46796	13.58653	13.39917
MIMAT0023713	hsa-miR-6088	11.85954	12.40758	13.05106	12.97859	12.34495	12.91993	12.9411	12.74061
MIMAT0023714	hsa-miR-6089	12.71975	12.78227	14.13672	14.10409	13.15344	13.71751	13.761	13.5851
MI0020366	hsa-miR-6089-1	10.22749	10.33042	11.08849	11.11791	10.8124	11.00324	10.48108	11.35228
MI0023563	hsa-miR-6089-2	10.22749	10.33042	11.08849	11.11791	10.8124	11.00324	10.48108	11.35228
MIMAT0023715	hsa-miR-6090	12.36707	12.7159	13.79054	13.71452	12.9851	13.60158	13.56417	13.43414
MIMAT0024598	hsa-miR-6125	11.84908	12.4234	13.19098	13.37919	12.28907	13.09798	13.13382	13.04671
MIMAT0024599	hsa-miR-6126	13.07052	13.11394	13.04422	13.11948	13.26395	12.54751	12.71868	13.64221
MIMAT0026622	hsa-miR-619-5p	7.765396	8.589596	7.49961	8.171875	7.425969	5.414082	5.716073	6.314283
MIMAT0003308	hsa-miR-638	11.61111	12.15508	12.85669	12.9007	12.11017	12.6658	12.63054	12.79147
MIMAT0003326	hsa-miR-663a	9.918096	10.77138	10.6467	11.34048	10.75367	10.48321	9.813308	11.13825
MIMAT0025854	hsa-miR-6722-3p	9.581276	10.37821	10.61702	11.11035	10.80873	10.04927	9.502072	10.98647
MIMAT0025856	hsa-miR-6724-5p	11.41892	11.78538	12.55269	12.48649	11.84866	12.32571	11.91957	12.3973
MIMAT0027355	hsa-miR-6727-5p	11.46937	12.15246	12.61482	12.80551	11.62491	12.47209	12.48954	12.52062
MIMAT0027359	hsa-miR-6729-5p	11.69064	12.33009	13.02769	13.11261	12.11539	12.95072	12.92046	12.88194

MIMAT0027365	hsa-miR-6732-5p	9.923204	10.539	8.921247	9.672468	10.13325	8.764148	7.802042	9.38286
MIMAT0027387	hsa-miR-6743-5p	10.64507	10.89444	11.10461	11.3571	10.98065	10.55639	10.09552	11.14869
MIMAT0027398	hsa-miR-6749-5p	9.237453	9.914562	10.42527	10.79861	10.39455	10.17741	9.477958	10.13375
MIMAT0027404	hsa-miR-6752-5p	10.76549	10.89462	11.53346	11.52007	11.32057	11.07624	10.67063	11.98253
MIMAT0027412	hsa-miR-6756-5p	9.403712	9.792755	9.644855	10.17177	10.10468	9.445856	9.010714	9.828904
MIMAT0027430	hsa-miR-6765-5p	10.47131	10.6816	11.03195	11.44527	11.13495	10.41797	9.629867	11.35712
MIMAT0027442	hsa-miR-6771-5p	8.1225	8.739004	8.972135	9.9052	8.667234	8.84818	8.036575	9.015805
MIMAT0027450	hsa-miR-6775-5p	9.572067	10.42916	10.34272	10.91984	10.30886	9.572145	9.02152	10.17669
MIMAT0027572	hsa-miR-6780b-5p	11.75482	11.91622	10.73194	11.11522	11.10522	9.570017	9.409493	11.12884
MIMAT0027472	hsa-miR-6786-5p	11.33813	11.97953	12.57088	12.41706	11.69014	12.03334	11.61347	12.28631
MIMAT0027474	hsa-miR-6787-5p	8.399772	10.00842	9.686008	10.45895	9.951094	8.108144	7.746926	10.31095
MIMAT0027478	hsa-miR-6789-5p	9.445251	9.830597	10.46406	10.97245	10.68609	10.53504	9.673205	10.90985
MIMAT0027480	hsa-miR-6790-5p	9.236917	8.22829	10.31651	10.44861	10.5115	9.887201	9.196329	10.35212
MIMAT0027482	hsa-miR-6791-5p	10.36947	10.61052	10.88165	11.49356	11.10232	11.06611	10.71453	11.90339
MIMAT0027488	hsa-miR-6794-5p	9.138513	9.823379	10.18729	10.76195	10.25851	9.212099	8.698524	10.55221
MIMAT0027496	hsa-miR-6798-5p	8.536935	8.968895	9.331532	10.43414	9.745407	8.819681	7.677833	10.58546
MIMAT0027498	hsa-miR-6799-5p	6.853444	6.999596	6.52441	7.699361	6.881133	6.370534	5.691249	7.087809
MI0022645	hsa-miR-6800	10.62243	10.87152	11.94068	11.81861	11.32287	11.57963	11.34759	11.60602
MI0022645	hsa-miR-6800	10.44979	10.77344	11.86325	11.75343	11.22407	11.5022	11.27804	11.51777

MIMAT0027500	hsa-miR-6800-5p	11.0751	11.21273	12.33736	12.35536	11.80794	12.30648	12.00056	12.5054
MIMAT0027506	hsa-miR-6803-5p	10.96534	11.34061	11.92045	12.09204	11.47014	11.42458	11.28293	11.95102
MIMAT0027510	hsa-miR-6805-5p	10.111	10.96665	10.97911	11.49443	10.78627	10.50226	9.949692	11.28382
MIMAT0027532	hsa-miR-6816-5p	10.32018	11.1339	11.40499	11.65324	11.12645	10.61195	10.33912	12.1631
MIMAT0027542	hsa-miR-6821-5p	10.77964	10.84896	11.64814	11.56919	11.40861	11.40488	11.11509	11.61541
MIMAT0027600	hsa-miR-6850-5p	10.49844	10.7079	11.37268	11.4697	11.15157	11.09479	10.72328	11.617
MIMAT0027616	hsa-miR-6858-5p	9.675191	10.3837	10.65373	11.04027	10.23827	10.4665	9.88053	10.55519
MIMAT0027638	hsa-miR-6869-5p	11.80603	12.46385	13.55843	13.03771	12.25934	13.28133	13.22785	12.62574
MIMAT0027658	hsa-miR-6879-5p	7.235421	8.515169	7.235695	8.247378	7.705547	6.190564	6.136084	7.672686
MIMAT0027682	hsa-miR-6891-5p	9.471685	9.920283	9.985174	10.3327	9.745407	9.260577	8.67315	7.812659
MIMAT0028111	hsa-miR-7107-5p	9.253173	9.642667	8.807929	9.767329	9.754754	7.685753	7.182767	10.3427
MIMAT0028113	hsa-miR-7108-5p	11.00166	11.31927	11.83818	12.25643	11.49228	11.91212	11.59356	12.37501
MIMAT0028117	hsa-miR-7110-5p	5.23844	7.006907	6.22165	7.08352	7.767258	6.165074	6.02357	7.267069
MIMAT0028211	hsa-miR-7150	10.67855	10.6342	10.77521	11.25774	10.85496	10.26606	9.562195	10.01741
MIMAT0010313	hsa-miR-762	11.04983	11.88189	12.09196	12.38078	11.46361	11.76138	11.45301	12.2794
MIMAT0030019	hsa-miR-7704	12.03147	12.78102	13.20771	13.45243	12.27396	13.20771	13.3381	12.88404
MIMAT0031180	hsa-miR-7977	9.873977	9.378177	11.38241	10.65163	8.08931	10.84908	10.37313	3.83727
MIMAT0030996	hsa-miR-8069	11.95441	12.36948	13.69442	13.62844	12.80941	13.42526	13.40679	13.12203
MIMAT0030999	hsa-miR-8072	12.24382	12.08357	12.09186	12.73783	12.27278	11.43734	11.27571	12.60635

MIMAT0031002	hsa-miR-8075	9.419784	10.50063	6.996563	8.78463	9.259838	6.243616	5.510298	6.511162
MIMAT0000092	hsa-miR-92a-3p	9.516838	9.697326	9.899425	9.892468	10.22702	10.28717	10.43872	10.70724
MIMAT0004792	hsa-miR-92b-5p	9.178785	10.55415	9.538787	10.56759	8.220593	9.219394	8.547501	9.419965
MIMAT0022938	hsa-miR-937-5p	7.7865	8.549046	9.170472	9.839099	9.287049	9.01414	8.223462	9.546305

The table lists miRNA accession numbers (miRBase), transcript IDs, and normalized expression values for UCMSC-EV Lot 1 through Lot 8.

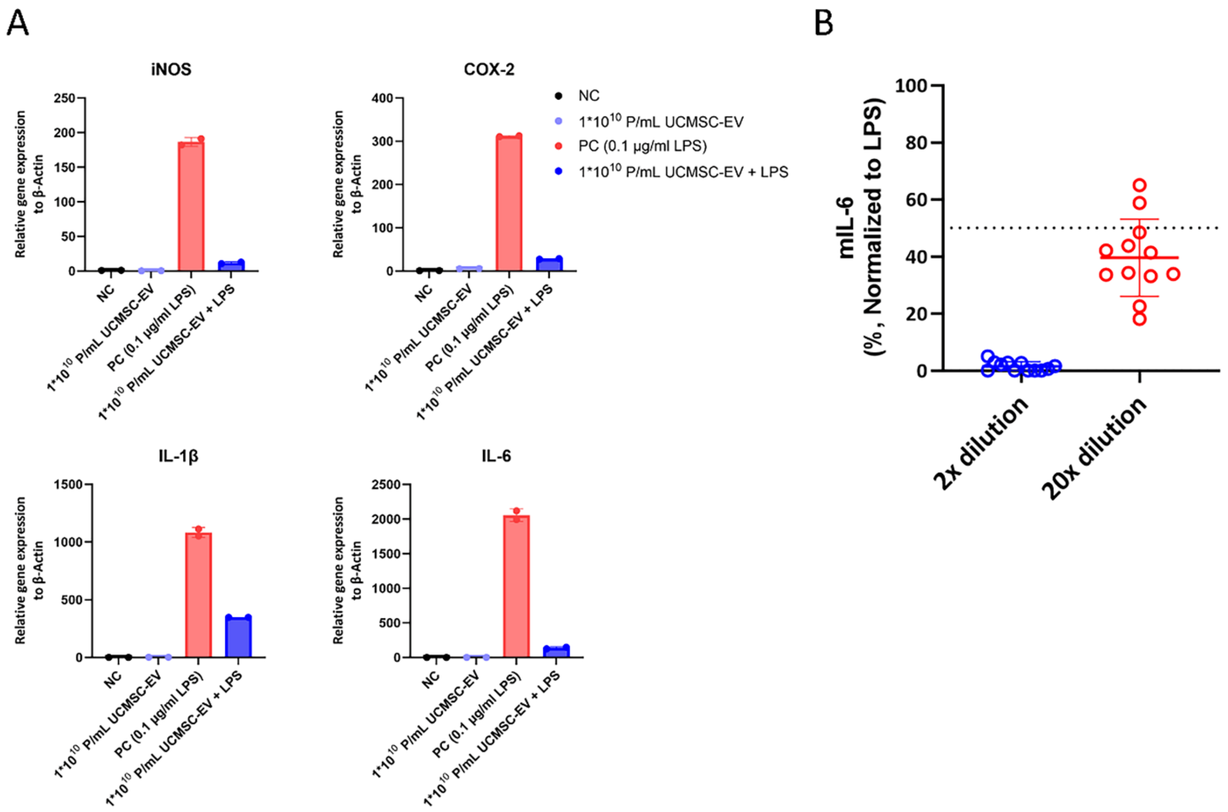

Supplementary Figure 1. UCMSC-EVs suppressed inflammatory effects in the LPS-induced RAW264.7 macrophage inflammatory assay. (A) The bar plot shows the inflammatory cytokines (COX-2, iNOS, IL-1β, IL-6) quantified by RT-qPCR. The results showed that UCMSC-EVs decreased LPS-induced inflammatory cytokines gene expression in RAW264.7 cells. Data are expressed as the mean ± standard deviation (SD) from two replicates of each condition; (B) The Dot plot represents relative mIL-6 protein levels in the LPS-induced RAW264.7 model. Data are presented as individual points with lines indicating mean ± SD. The horizontal dotted line denotes the 50% response/inhibition threshold.

RAW264.7 cells were obtained from BCRC (BCRC number: 60001, Taiwan). Cells were maintained and grown in DMEM high glucose medium (10566016, Gibco) supplemented with 10% fetal bovine serum (A5256701, Gibco), and 1% Antibiotic-Antimycotic (100X) (15240062, Gibco). RAW264.7 cells were seeded into 24-well plates at $1x10^5$ cells per well, and incubated at 37 °C in a CO_2 incubator for 24 hours. After cell adhered, RAW264.7 cells were induced with 0.1 μg/mL LPS in the presence or absence of UCMSC-EVs for 24 hours. The gene expression of the induced inflammatory cytokines (iNOS, COX-2, IL-1β, IL-6) were quantified by RT-qPCR. Total cell RNA was extracted using Quick-RNA™ MiniPrep Kit (R1055, ZYMO Research) following the manufacturer's instructions. The RT-qPCR was performed with Power SYBR™ Green RNA-to-CT™ 1-Step Kit (4389986, ThermoFisher) on QuantStudio™ 5 Real-Time PCR System (ThermoFisher, USA). The relative expression of all mRNAs was normalized to β-Actin. The data were calculated using the $2^{-\Delta\Delta Ct}$ method. The primer sequences are detailed in the Table below.

Table. Forward and reverse primers used in the LPS-induced RAW264.7 macrophage inflammatory cytokine RT-qPCR study

Primer	Primer sequence (F)	Primer sequence (R)
Mouse_β-actin	CATTGCTGACAGGATGCAGAAGG	TGCTGGAAGGTGGACAGTGAGG
Mouse_iNOS	TGAAGAAAACCCCTTGTGCT	TTCTGTGCTGTCCCAGTGAG
Mouse_COX-2	GAAGATTCCCTCCGGTGTTT	CCCTTCTCACTGGCTTATGTAG
Mouse_IL-1β	AGGTCAAAGGTTTGGAAGCA	TGAAGCAGCTATGGCAACTG

Mosue_IL-6	TCTGAAGGACTCTGGCTTTG	GATGGATGCTACCAAACTGGA

COX-2: cyclooxygenase-2; IL-1β: interleukin-1 beta; IL-6: interleukin-6; iNOS: inducible nitric oxide synthase.

To evaluate the batch-to-batch anti-inflammatory potency of UCMSC-EVs, two concentrations of UCMSC-EVs, 2× and 20× volume-based dilutions, were tested in the established LPS-induced RAW264.7 macrophage model. Secreted IL-6 levels in the supernatants were quantified using a mouse IL-6 ELISA kit (ELM-IL6-1, RayBiotech) according to the manufacturer's protocol. For comparative analysis, IL-6 concentrations were normalized to the LPS-only positive control (PC: 100%) and the naïve negative control (NC: 0%). As anticipated, a dose-dependent anti-inflammatory response was observed: the higher concentration (2× dilution) demonstrated potent inhibition (> 80%), while the lower concentration (20× dilution) maintained moderate activity, with most lots achieving > 50% inhibition. These results suggest that large-scale (20L) manufactured UCMSC-EVs robustly suppress inflammatory signaling.
